# Genomic Assessment of Potential Probiotic *Lactiplantibacillus plantarum* CRM56-2 Isolated from Fermented Tea Leaves

**DOI:** 10.21315/tlsr2024.35.2.12

**Published:** 2024-07-31

**Authors:** Engkarat Kingkaew, Naoto Tanaka, Yuh Shiwa, Jaruwan Sitdhipol, Rattanatda Nuhwa, Somboon Tanasupawat

**Affiliations:** 1 Department of Biochemistry and Microbiology, Faculty of Pharmaceutical Sciences, Chulalongkorn University, Bangkok 10330, Thailand; 2 Department of Biology, School of Science, King Mongkut’s Institute of Technology Ladkrabang, Bangkok 10520, Thailand; 3 Department of Molecular Microbiology, Faculty of Life Sciences, Tokyo University of Agriculture, 1-1-1 Sakuragaoka, Setagaya-ku, Tokyo 156-8502, Japan; 4 Biodiversity Research Centre, Research and Development Group for Bio-Industries, Thailand Institute of Scientific and Technological Research, Pathum Thani 12120, Thailand

**Keywords:** *Lactiplantibacillus plantarum*, Fermented Tea Leaves, Genome Sequencing, Safety Evaluation, Probiotic Properties, Cholesterol-Lowering Activity

## Abstract

*Lactiplantibacillus plantarum* is a widely studied species known for its probiotic properties that can help alleviate serum cholesterol levels. Whole-genome sequencing provides genetic information on probiotic attributes, metabolic activities and safety assessment. This study investigates the probiotic properties of strain CRM56-2, isolated from Thai fermented tea leaves, using Whole-Genome Sequencing (WGS) to evaluate the safety, health-promoting genes and functional analysis. Strain CRM56-2 showed bile salt hydrolase (BSH) activity, assimilated cholesterol at a rate of 75.94%, tolerated acidic and bile environments and attached to Caco-2 cells. Based on ANIb (98.9%), ANIm (99.2%), and digital DNA–DNA hybridisation (98.3%), strain CRM56-2 was identified as *L. plantarum*. *In silico* analysis revealed that it was not pathogenic and contained no antibiotic-resistance genes or plasmids. *L. plantarum* CRM56-2 possessed genes linked to several probiotic properties and beneficial impacts. The genome of strain CRM56-2 suggested that *L. plantarum* CRM56-2 is non-hazardous, with potential probiotic characteristics and beneficial impacts, which could enhance its probiotic application. Consequently, *L. plantarum* CRM56-2 demonstrated excellent cholesterol-lowering activity and probiotic properties.

**Highlights**

- Strain CRM56-2 isolated from fermented tea leaves was identified as *Lactiplantibacillus plantarum* based on ANIb (98.9%), ANIm (99.2%), and digital DNA–DNA hybridisation (98.3%).
- *L. plantarum* CRM56-2 showed bile salt hydrolase (BSH) activity, assimilated cholesterol at a rate of 75.94%, tolerated acidic and bile environments, and attached to Caco-2 cells.
- Based on genomic analysis, *L. plantarum* CRM56-2 possessed genes linked to several probiotic properties and health-promoting effects, which could enhance its probiotic application.

## INTRODUCTION

Lactic acid bacteria (LAB) are commonly used as probiotics because of their desirable features, such as safety, and longevity in the gastrointestinal tract (GIT) (Ye *et al*. 2020). *Lactobacillus plantarum* is mostly versatile of the extensively studied species, and it is found in fermented meat and plant sources (Siezen *et al*. 2010). The ability of *L. plantarum* to survive gastric and bile and its ability to survive and propagate in the GIT (Le & Yang 2018; Zhang *et al*. 2020) make it a promising target for probiotic research. In addition, *L. plantarum* is receiving attention in pharmaceutical sciences due to its cholesterol-lowering properties (Arasu *et al*. 2016). Probiotics can lower serum cholesterol using prebiotics to synthesise short-chain fatty acids (SCFAs), which can inhibit hepatic cholesterol synthesis, decrease serum lipids, and directly assimilate cholesterol (Pereira & Gibson 2002).

Several *L. plantarum* strains show potential probiotic traits, including adaptable growing characteristics, stress endurance, potent GIT survival and physiological roles, such as cholesterol-lowering (van den Nieuwboer *et al*. 2016). However, safety assessment is crucial in food and health applications, considering the growing concerns regarding antibiotic resistance and virulence factors. Therefore, whole-genome sequencing (WGS) analysis enables precise identification while providing molecular information on probiotic attributes, putative metabolisms and safety evaluation, such as virulent genetic elements, antibiotic resistance genes (ARGs), as well as genetic factors associated with risky substances (Guinane *et al*. 2016; Li *et al*. 2017). With the publication of whole-genome sequences of several *L. plantarum* strains in the NCBI database, comprehensive knowledge of *L. plantarum*’s functional properties and innovative applications becomes achievable. The genomic analysis must define a probiotic characteristic of an interesting strain. Selection criteria were bile salt hydrolase (BSH) activity, the highest cholesterol assimilation ability, and one of the significant probiotic species. Furthermore, genomic characterisation of *L. plantarum* strains isolated from the Thai-fermented leaves of *Camellia sinensis* needs investigation.

Accordingly, the present study endeavored to assess both the cholesterol-lowering effects and probiotic properties of strain CRM56-2. Additionally, WGS was employed to appraise the safety and probiotic-associated genes of this strain CRM56-2.

## MATERIALS AND METHODS

### Isolation

Strain CRM56-2 was isolated from *Camellia sinensis* obtained from the Chiang Rai province in Thailand. The sample (0.5 g) was added in De Man, Rogosa and Sharpe (MRS) broth and incubated at 37°C for 48 h–72 h. A loopful of the culture was streaked on MRS agar plates containing 0.3% (w/v) CaCO_3_. A single colony surrounded by a clear zone was selected and purified on MRS agar plates. The pure culture was preserved in 10% skim milk at −80°C and lyophilised.

### Identification

#### Phenotypic characteristics

The strain’s cell shape, size, arrangement and colonial appearance were observed by growing cells on MRS agar plates for two days. Gram staining was performed following the procedure described by Cowan and Steel (1965). The activity of catalase, reduction of nitrate, gas formation, hydrolysis of arginine, aesculin production, as well as slime formation were determined using the methods previously reported by Tanasupawat *et al*. (2002). Growth under different pH (3.5–10.0), temperatures (10, 15, 30, 37, 40, 42 and 45°C), and NaCl concentrations (1, 3, 5, 6, 6.5, 7, 7.5, and 8% w/v) were evaluated in MRS broth. Acid production from carbohydrates was determined following the methods reported by Tanasupawat *et al*. (2002). The lactic acid isomer was analysed using the enzymatic method described by Okada *et al*. (1978).

#### 16S rRNA gene sequencing analysis

The initial species identification of strain CRM56-2 was carried out using the 16S rRNA gene. It was amplified following the protocol by Phuengjayaem *et al*. (2017) and sequenced using universal primers [27F (5′-AGAGTTTGATCMTGGCTCAG-3′) and 1492R (5′TACGGYTACCTTGT-TACGACTT′3)] as described by Lane (1991) on a sequencer (Macrogen, Korea). Similarities of the 16S rRNA gene of strain CRM56-2 to the database were enumerated using the Ezbiocloud web-based tools (Yoon *et al*. 2017). The data were deposited in the DNA Data Bank of Japan (DDBJ), Mishima, Japan. The DDBJ accession number of strain CRM56-2 was LC742934.

#### Genomic sequencing, assembly and features

The genomic DNA was extracted following the procedure outlined by Yamada and Komagata (1970). Subsequently, the preparation of the library and sequencing were carried out at the Faculty of Life Sciences, Tokyo University of Agriculture, utilising the Nextera DNA Flex Library Prep Kit and the Illumina MiSeq platform with MiSeq v3 reagent kit (600 cycles). The genomic quality was determined using FastQC web-based tool, and TrimGalore web-based tool was applied to remove adaptors and low-quality reads. Filtered Illumina reads were assembled using Unicycler (Galaxy Version 0.4.8.0), and CheckM was used to evaluate the genomic quality (Park *et al*. 2015). The JSpeciesWS online server tool (Richter & Rosselló-Móra 2009; Richter *et al*. 2016) and Genome-to-Genome Distance Calculator (GGDC 2.1) (Meier-Kolthoff *et al*. 2013) were used to examine the average nucleotide identity (ANI) and digital DNA-DNA hybridisation (dDDH) data. TYGS web server (https://tygs.dsmz.de/) was used to construct the phylogenomic tree (Meier-Kolthoff & Göker 2019). Furthermore, the circular genomic map was generated using the Proksee Server (Stothard *et al*. 2019).

#### Gene annotation and functional prediction

The DFAST server (Tanizawa *et al*. 2018), Rapid Annotation Server Technology (RAST) (Aziz *et al*. 2008), PATRIC (Davis *et al*. 2020), and the NCBI Prokaryotic Genome Annotation Pipeline (PGAP) (Tatusova *et al*. 2016) were utilised to annotate the draft genome. PlasmidFinder (Carattoli *et al*. 2014) was used to detect plasmid. Genomic features are listed in Table 1. ResFinder (Bortolaia *et al*. 2020) was utilised to investigate antibiotic resistance genes. PathogenFinder was applied to predict pathogenicity (Cosentino *et al*. 2013). Putative prophage sequences were annotated and determined using the PHAge Search Tool Enhanced Release (PHASTER) (Arndt *et al*. 2016). The dbCAN meta server (https://bcb.unl.edu/dbCAN2/blast.php) with HMMER: biosequence analysis with profile hidden Markov models (version: 3.3.2) was used to identify carbohydrate-active enzymes, and all data produced by dbCAN depended on the CAZy database’s family classification (http://www.cazy.org/) (Cantarel *et al*. 2009; Zhang *et al*. 2018). The pathways and genes of strain CRM56-2 were examined and annotated using the Kyoto Encyclopedia of Genes and Genomes (KEGG) database (Kanehisa *et al*. 2016).

### *In vitro* Probiotic Assays

#### Cell suspension preparation

Strain CRM56-2 was cultured in MRS broth for 24 h at 30°C. Subsequently, the cell-free supernatant was removed by centrifugation at 14,000 rpm for 10 min at 4°C. The cells were washed with phosphate buffer (0.1 M, pH 7.2) and resuspended in phosphate buffer (0.1 M, pH 7.2) to obtain a cell suspension of 10^9^ CFU/mL.

#### BSH activity

The Kingkaew *et al*. (2022) approach was utilised to assess BSH activity. Taurodeoxycholic acid sodium salt (TDCA) [0.5% (w/v)] and calcium chloride (CaCl_2_) [0.037% (w/v)] were added to MRS agar medium. A CRM56-2 cell suspension was spotted onto the agar, and the plates underwent a 72 h anaerobic incubation period at 37°C. Halos or opaque white colonies with halos around them suggested BSH activity. The control was MRS agar containing no TDCA and CaCl_2_.

#### Assimilation of cholesterol

Using MRS broth supplemented with cholesterol-polyethylene glycol (PEG) 600 (Sigma, India) at a final concentration of 100 μg/mL, the capacity of strain CRM56-2 to absorb cholesterol was assessed. The suspension of strain CRM56-2 (1%, v/v) was added into the MRS broth containing cholesterol-PEG 600 and incubated at 37°C for 24 h under anaerobic conditions. Following the procedure of Tomaro-Duchesneau *et al*. (2014), the cholesterol was isolated, and the concentration of cholesterol was quantified using the technique described by Rudel and Morris (1973). The cholesterol concentration was compared to a reference curve prepared using a cholesterol stock solution. The ability to assimilate cholesterol was determined by calculating the percentage of assimilated cholesterol (%) at each incubation, as follows:


(1) $$\text{Assimilated cholesterol }(\mu \text{g}/\text{mL})=[\text{Cholesterol }{\left(\mu \text{g}/\text{mL}\right)}_{\left(0\;\text{h}\right)}-\text{Cholesterol }{\left(\mu \text{g}/\text{mL}\right)}_{\left(24\;\text{h}\right)}]$$

(2) $$\text{Assimilated cholesterol }(\%)=\frac{\text{Assimilated cholesterol }(\mu \text{g}/\text{mL})}{\text{Cholesterol }(\mu \text{g}/\text{mL})(0\;h)}\times 100$$

#### Acid and bile tolerance properties

The acid and bile tolerance properties of strain CRM56-2 were determined according to the method of Hyronimus *et al*. (2000). For acid tolerance, 2% of LAB suspension (10^9^ CFU/mL) was inoculated into MRS broth with a pH of 3.0 and 6.5, and incubated anaerobically at 37°C for 180 min. The samples were collected at 0 min and 180 min of incubation time to enumerate viable cells. For bile salt tolerance, 2% of LAB suspension (10^9^ CFU/mL) was inoculated into MRS broth with a pH of 8.0, containing bile salt (0.3% (w/v)), as well as without supplementation of bile salt, and incubated anaerobically at 37°C for 3 h. The samples were collected at 0 min and 180 min of incubation time to count the remaining viable cells. The results were described as colony-forming units per milliliter (CFU/mL).

#### The ability of adhesion to Caco-2 cells

The adhesion ability of strain CRM56-2 to adhere to the intestinal epithelium was evaluated following the method reported by Bustos *et al*. (2012). Briefly, Caco-2 cells were inoculated into 24-well tissue culture plates at a concentration of 5 × 10^5^ cells/mL and incubated at 37°C with 5% CO2 for 15 days, with culture medium changes every 72 h. Overnight cultures of strain CRM56-2 in MRS broth were collected by centrifugation at 14,000 rpm for 10 min at 4°C and cleansed with phosphate buffer solution. Subsequently, the strain CRM56-2 cells (10^9^ CFU/mL) were resuspended in DMEM supplemented with 10% fetal bovine serum (FBS) and inoculated onto the Caco-2 cells in each well, followed by incubation at 37°C with 5% CO2 for 90 min. The cells were washed thrice with PBS and lysed with 0.05% (v/v) Triton-X solution. The released bacterial cells were serially diluted, spotted onto MRS agar, and incubated at 37°C for 2 days. The adhesive capability was expressed as the percentage of adhesive cells to Caco-2 cells to the total sum of bacteria (CFU/mL). *Lacticaseibacillus rhamnosus* GG was used as a positive control.

## RESULTS AND DISCUSSION

### Strain Identification

Strain CRM56-2 is a Gram-positive, catalase-negative, facultatively anaerobic rod. It synthesises DL-lactic acid from D-glucose homofermentative and does not produce gas from glucose. The strain can grow at a temperature range of 15°C–45°C, a pH range of 2–9, as well as in the presence of NaCl (1%–6% (w/v)). Strain CRM56-2 cannot hydrolyse arginine or reduce nitrate and does not form slime. Acid is generated from various sugars such as D-arabinose, D-cellobiose, D-fructose, D-galactose, D-glucose, lactose, D-mannose, D-maltose, D-mannitol, D-melibiose, D-raffinose, L-rhamnose, D-ribose, salicin, D-sorbitol, D-saccharose, D-trehalose and D-xylose. Using the entire sequences of the 16S rRNA gene(1,567 bp), strain CRM56-2 was closely related to *L. paraplantarum* DSM 10667^T^,*L.pentosus* DSM 20314^T^, *L. plantarum* ATCC 14917^T^ and *L. argentoratensis* DSM16365^T^, with a similarity of 99.73%, 99.93%, 100% and 100%, respectively.

**Figure 1:**
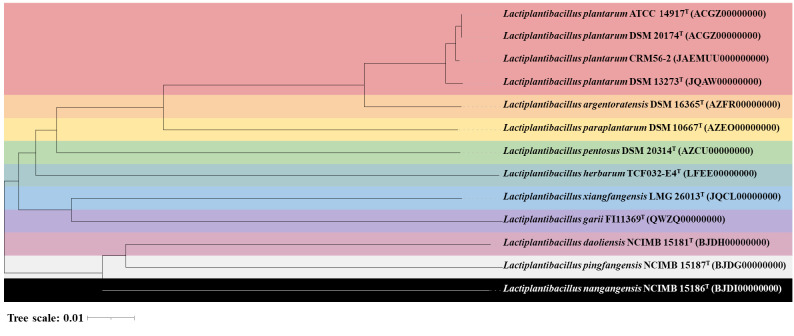
The phylogenomic tree was generated using whole genome sequencing data from strain CRM56-2 and closely related type strains.

Based on the phylogenomic tree (Fig. 1), strain CRM56-2 was grouped with various strains of *L. plantarum*. A dDDH score of 98.3% was observed between strain CRM56-2 and *L. plantarum* DSM ATCC 14917^T^ and/or 20174^T^, which was the highest dDDH value among closely related species (Table 2). Furthermore, this strain exhibited the highest ANIb and ANIm values of 98.92% and 99.27%, respectively, to *L. plantarum* ATCC 14917^T^ (Table 2). The ANI and dDDH values, which were higher than the species boundary value (ANI > 95%–96%), confirmed that strain CRM56-2 was unequivocally identified as *L. plantarum* (Chun *et al*. 2018). Therefore, conventional tests and genomic investigations confirm that strain CRM56-2 belongs to *L. plantarum*.

### Genomic Features of Strain CRM56-2

**Figure 2:**
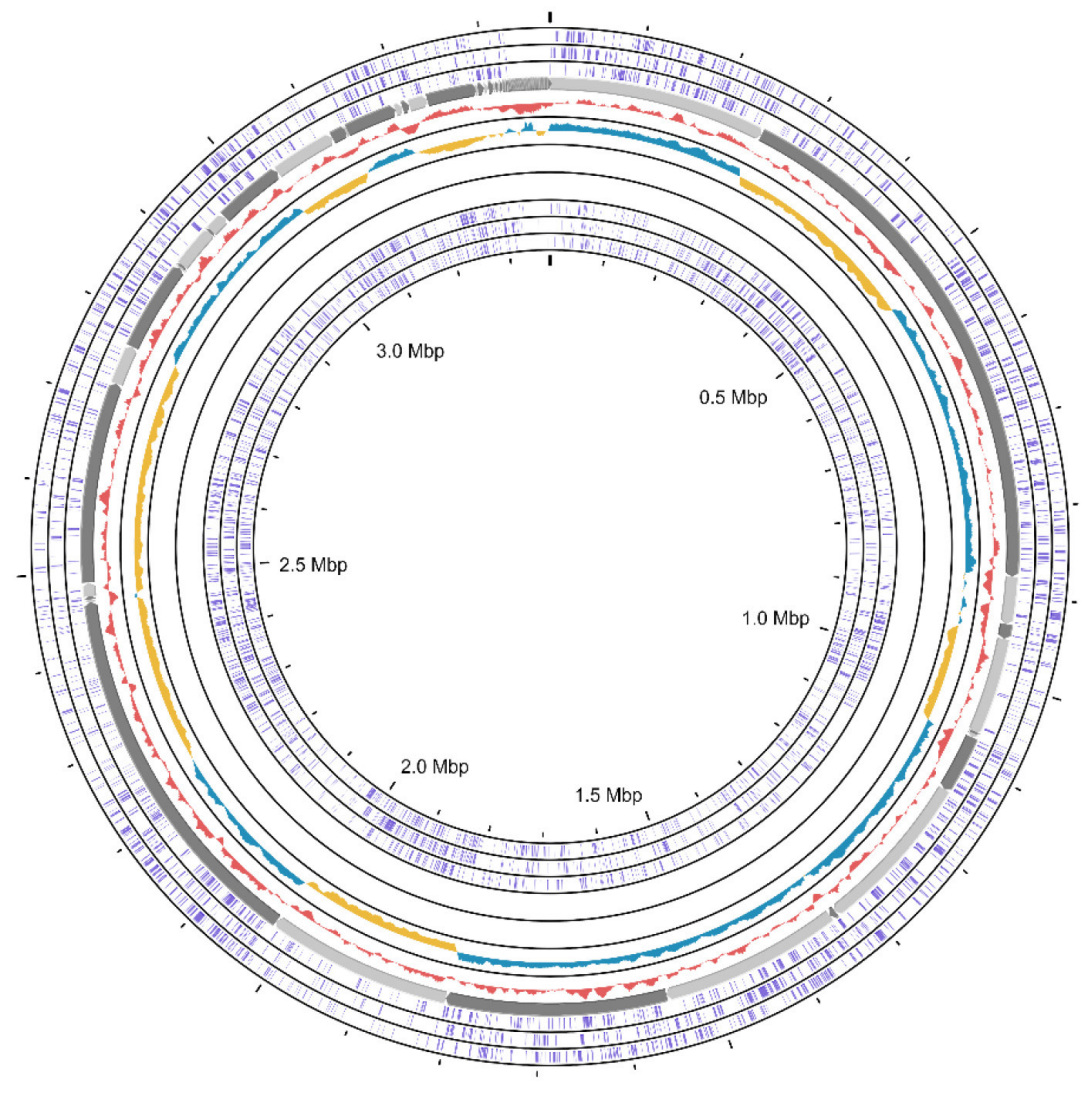
Circular genomic map of *L. plantarum* CRM56-2. The information is indicated as follows: Open reading frames (ORFs) (purple), GC skew (+) (blue), GC skew (–) (yellow) and GC content (pink).

Based on Table 1, *L. plantarum* CRM56-2 (JAEMUU00000000) had a genome size of 3,373,611 bp with an N50 of 216,722, L50 of 5, and a genome coverage of 437×. The DNA G+C content of CRM56-2 was 44.3%. The genome sizes and G+C content fell within the 3 Mb–3.6 Mb range and 44%–45%, respectively. These values are consistent with previous reports for this species (Martino *et al*. 2016; Surve *et al*. 2022). This finding represents the inaugural report of *L. plantarum* isolation from a fermented plant source in northern Thailand. PGAP annotation reported 3,270 genes, including 3,079 coding genes, 111 pseudogenes, 80 RNA genes, 71 tRNAs and 4 ncRNAs. The genomic statistics are shown in Table 3. Strain CRM56-2 lacked CRISPRs and Supplementary Material Fig. 1 illustrates its subsystems. Fig. 2 shows the circular genome of strain CRM56-2.

## CONCEIVABLE GENE FACTORS IN PROTEASE ACTIVITIES, METABOLISM OF CARBOHYDRATES AND BENEFICIAL METABOLITES OF *L. PLANTARUM* CRM56-2

Enzymes-associated genes with conserved proteolytic and metabolic sugar systems could be found in strain CRM56-2, allowing for the performance of a functional genomic investigation. The genome of strain CRM56-2 encodes several proteases, such as peptidases (*pep*), proteinase (*prt*), and an oligopeptide ABC transport system (*opp*). Peptidase enzymes cleave various compounds, including asparagine, casein, cysteine, glutamate-derived peptides, leucine, methionine, proline and serine (De Jesus *et al*. 2022) (Refer Supplementary Material Table S1 for details).

Various enzymes related to the breakdown and utilisation of carbohydrates, such as chitobiose, fructose, galactose, glucose, mannose and sucrose, were identified in the genome of *L. plantarum* CRM56-2. These include 6-phospho-beta-glucosidase, glucokinase, phosphoglucomutase and phosphomannose isomerase. Moreover, genes involved in transporting cellobiose, fructose, glucose and mannose, mainly through the PTS system, were also discovered (De Jesus *et al*. 2022) (Refer Supplementary Material Table S2 for details).

Furthermore, the analysis of carbohydrate-active enzymes (CAZymes) genes in the *L. plantarum* CRM56-2 genome revealed that they belong to carbohydrate-binding modules (CBMs) (CBM32, CBM34, CBM48) (*n* = 3), glycoside hydrolases (GHs) (GH1, GH2, GH13, GH25, GH31, GH32, GH36, GH38, GH42, GH65, GH70, GH73, GH78, GH85, GH126, GH170) (*n* = 48), and glycosyltransferases (GTs) families (GT2, GT4, GT5, GT26, GT28, GT32, GT35, GT51) (*n* = 31) (see Supplementary Material Table S4 for details). The high number and diversity of CAZyme genes suggests that *L. plantarum* CRM56-2 can utilise a variety of monosaccharides and polysaccharides as energy sources and synthesise molecules. Notably, prebiotics associated with human gut health and found in oligosaccharides are degraded by GH13 and GH32. The GH families also play a key role in oligosaccharide synthesis, indicating that strain CRM56-2 and other probiotics may be used as prebiotics (Abriouel *et al*. 2017). In addition, GTs expedite the transfer of sugars from activated donor molecules to certain acceptors, they are necessary to produce structural surfaces that the host immune system is able to recognise (Mazmanian *et al*. 2008).

In summary, the abundance and diversity of CAZymes genes in *L. plantarum* CRM56-2 suggest that this strain has a strong potential for immunomodulation and pathogen prevention as a probiotic.

Several genes encoding essential enzymes involved in the fermentation process were identified in *L. plantarum* CRM56-2, including acetate kinase, glyceraldehyde 3-phosphate dehydrogenase, glucose-6-phosphate isomerase, glucokinase, phosphoglycerate kinase, phosphoketolase, pyruvate kinase, pentose-5-phosphate 3-epimerase, lactic acid dehydrogenase and others (De Jesus *et al*. 2022). These enzymes play a critical role in the production of acetate or lactate (De Jesus *et al*. 2022). The study also investigated genes associated with vitamin production, such as dihydrofolate reductase (B9), riboflavin kinase (B2), and thiamine pyrophosphokinase (B1), as well as butyrate-associated genes (Supplementary Material Table S3 for details) (Botta *et al*. 2017). KEGG annotation revealed butanoate metabolism in *L. plantarum* CRM56-2, with genes implicated in butyric acid synthesis linked to the complimentary functions of a medium-chain thioesterase and FASII in CRM56-2.

These metabolic characteristics help the CRM56-2 strain ferment substances and produce useful metabolites such bioactive peptides, lactate, SCFA and vitamins. Key enzymes include acetate kinase, glyceraldehyde-3-phosphate dehydrogenase, lactate dehydrogenase, phosphoketolase, peptidases, proteinases, pyruvate kinase, thiamine pyrophosphokinase and riboflavin kinase are associated with the Embden-Meyerhof (EMP) or phosphoketolase pathways and proteolysis in strain CRM56-2. Moreover, the study investigated butyric acid production-associated genes linked to the complementary activities of the FASII pathway and the medium-chain acyl-ACP thioesterase. Based on these findings, the presence of these metabolic-associated genes was similar to the other *L. plantarum* strains (Botta *et al*. 2017). These findings could have illustrated a previously unexplained metabolic pathway for the production of butyric acid in *L. plantarum*. Furthermore, these beneficial bioproducts are helpful in GIT inflammation.

### Cholesterol-Lowering Activities

This study assessed the BSH activity BSH and cholesterol assimilation ability of strain CRM56-2 to evaluate its hypocholesterolemic effects (Table 4).

### BSH and Cholesterol Assimilation

This study examined the BSH activity BSH and cholesterol assimilation ability of strain CRM56-2 to assess its hypocholesterolemic effects. The strain exhibited BSH activity, confirmed by the choloylglycine hydrolase (*bsh*) gene, and demonstrated the ability to assimilate cholesterol at 75.94%. These properties indicate the presence of cholesterol-lowering effects on the host, making BSH activity a desirable probiotic characteristic according to the FAO/WHO Guidelines for the Evaluation of Probiotics in Food (FAO/WHO 2002). However, deconjugated bile due to BSH activity may pose safety concerns, as it can negatively impact lipid digestion, disrupt intestinal environments, lead to gallstones, and be converted to carcinogens. Fortunately, CRM56-2 lacked genes to generate secondary bile salts, indicating no safety concerns related to hazardous secondary bile compounds.

The genome analysis of strain CRM56-2 showed cholesterol assimilation-associated genetic elements, including *fba, ccpA* and *glgP*. The capacity of the strain to absorb cholesterol is explained by the genes linked with cholesterol assimilation, which encode membrane-related proteins that can bind to the cholesterol molecule and further integrate it into the cell. LAB may absorb cholesterol from the GIT by attaching to the surface and utilising putative enzymatic activities, affecting the process of cholesterol absorption (Kingkaew *et al*. 2023). The cholesterol-lowering effects-associated genes are summarised in Table 5. In conclusion, strain CRM56-2’s capacity to deconjugate bile salts and assimilate cholesterol makes it a promising probiotic candidate with potential hypocholesterolemic effects on the host.

### *In vitro* Probiotic Properties

#### Acid and bile tolerance and adhesion ability

For the assessment of acid tolerance, strain CRM56-2 was inoculated in MRS broth with a pH of 3.0 and incubated for 180 min. The viability of the strain was reduced from 2.4 × 10^7^ to 5.8 × 10^6^ CFU/mL. To evaluate bile tolerance, strain CRM56-2 was incubated in MRS broth supplemented with 0.3% bile salt at pH 8.0 for 3 h. The viability of the strain slightly increased from 2.7 × 10^6^ to 7.7 × 10^6^ CFU/mL (Table 3). Furthermore, the adhesion ability of strain CRM56-2 and *L.rhamnosus* GG was found to be approximately 0.40 ± 0.15% and 0.40 ± 0.05%, respectively.

### Probiogenomic Characteristics and Safety Assessment

#### Probiogenomic characteristics

The genome of *L. plantarum* CRM56-2 contains encoded genes associated with stress response in the GIT and adhesion ability, including ATP-dependent ClpX protease, chaperones (*GroeL, DnaJ, DnaK*), enolase, F_0_F_1_ ATP system genes, Na^+^/H^+^ antiporter NhaC, glycine/betaine ABC transporter permease, S-ribosylhomocysteine lyase, two-component sensor histidine kinase, ornithine decarboxylase, serine protease HtrA, and others, as shown in Table 6. Probiotictraits are strain-specific, motivating searching for new superior strains. *L. plantarum* CRM56-2 showed excellent tolerance to acid and bile salt conditions. Additionally, analysis of the strain’s genome indicated that its proteinaceous compounds play a role in environmental and genetic processing information and metabolic function, suggesting the relevance of these genes in maintaining the biological function of strain CRM56-2 in specific contexts or hosts. The strain’s core genome contained genes involved in stress response mechanisms, such as bile efflux and proton extrusions, metabolic response, heat shock/chaperones protein synthesis expression and transcriptional regulators. These genetic components may be crucial to the bacterial endurance of strain CRM56-2 in the human GIT. Probiotic activity requires adhesion to the intestinal mucosa epithelium, and microbial surface proteins have been linked to colonisation (Ye *et al*. 2020). Genes encoding cell-surface proteins, such as elongation factor Tu and lipoprotein signal peptide, were found in *L. plantarum* CRM56-2. The strain also contained a gene encoding LPXTG-specific sortase, relevant to strain adherence to surrounding epithelial tissue. Cell-surface proteins known as sortase-dependent proteins are crucial for adhesion (Alayande *et al*. 2020). The adhesion-associated genes discovered in strain CRM56-2 may thus improve strain stability and aid in successful colonisation.

#### Safety assessment (pathogenicity, antibiotic resistance genes and mobile genetic elements)

After a comprehensive genome analysis, the strain CRM56-2 was identified and predicted to be a non-pathogenic microorganism (Table 7). The genome lacked mobile genetic elements such as plasmids and ARGs (Tables 1 and 7). Additionally, six prophage regions, including two intact prophages ranging in size from 29.5 to 44 Kb, three incomplete prophages (5.4 to 23.3 Kb), and one questionable prophage (18.8 Kb), were integrated into the chromosome (Supplementary Material Table S5 for details). Although virulent genetic elements were present, including the capsular polysaccharide biosynthesis protein (*cps4E*) and exopolysaccharides biosynthesis proteins (*cps4B* and *cps2B*), the genome of the strain CRM56-2 contained no ARGs. It was identified as a non-human pathogen, indicating that the strain is safe and minimises the chance of spreading ARGs to the host’s gut normal flora.

Phages are frequently found in the genomes of *Lactobacillus* species (probiotic), but no virulence factors or genes associated with pathogenic qualities were found in these phage regions. Prophages may improve bacterial fitness in unfavourable environments (Pei *et al*. 2021). Capsular- and exo-polysaccharide genes associated with virulence elements were explored. Exopolysaccharides improve the capacity of bacteria to survive under oxidative and osmotic stress and contribute to its ability to adhere (Diale *et al*. 2021). Additionally, commercial strains like the accepted Generally Recognised as Safe (GRAS) *L. plantarum* 299V contained the hemolysin III gene (*hlyIII*), as well as a number of *Lactobacillus* strains. If no additional virulence genes have been found in the genome, strains with the *hlyIII* gene are often regarded as benign. Numerous studies have been conducted on hemolysin III and its harmlessness in lactobacilli. Therefore, this proteinaceous compound is not a serious concern (Surachat *et al*. 2017). Because they increase bacterial survival, these genes are advantageous to the bacterium and may be required when viable cells are needed.

## CONCLUSION

In this study, *L. plantarum* CRM56-2 was isolated from *Camellia sinensis* and found to express BSH activity, which was indicated by the development of an opaque white colony. Additionally, CRM56-2 could withstand acidic and bile salt environments, metabolise cholesterol by over 70%, and possibly attach to Caco-2 cells. The genomic assessment of *L. plantarum* CRM56-2 highlighted its appeal as a promising probiotic. The strain was deemed harmless due to the absence of ARGs, plasmids, and virulent genetic features. Furthermore, the strain contains several genes involved in the tolerance of acid and bile salts, adhesive ability, and other beneficial impacts. Based on the *in vitro* and *in silico* investigations, it was concluded that strain CRM56-2 has health-promoting benefits and probiotic features, making it a promising probiotic. The genetic information of this strain supported its favorable traits.

## Supplementary Information



## Figures and Tables

**Table 1:** Genomic features of *Lactiplantibacillus plantarum* CRM56-2 and *L. plantarum* 299V.

Attribute(s)	CRM56-2	299V
Source	Fermented tea leaves	Healthy human intestinal mucosa
Accession no.	JAEMUU000000000^F^	LEAV00000000^F^
Genome size (bp)	3,373,611^c^	3,302,055^c^
Plasmids	0^E^	2 (rep28, 98.17% identity; rep38, 99.0% identity)^E^
Genome qualities:		
- Genome quality	Good^a^	Good^a^
- Completeness (%)	100^b^	99.35^b^
- Coarse consistency	98.2^a^	98^a^
- Fine consistency	95.6^a^	96.2^a^
G+C content (%)	44.3^c^	44.4^c^
Genome coverage	437x^f^	48x^f^
N50	216,722^c^	173,004^c^
L50	5^c^	8^c^
No. of contig	204^c^	67^c^
No. of subsystem	235^c^	232^c^
No. of coding	3,449^c^	3,264^c^
sequences		
No. of RNA	73^c^	60^c^
No. of CRISPRS	0^D^	0^D^

*Notes*:

a = Data obtained from PATRIC;

b = Data obtained from CheckM;

c = Data obtained from RAST web-based tool;

D = Data obtained from DFAST annotation;

E = Data obtained from PlasmidFinder;

F = Data obtained from NCBI.

**Table 2:** ANIb and ANIm (%) and the digital DNA-DNA hybridisation (dDDH) values between the draft genomes of the strain CRM56-2; *L. plantarum* DSM ATCC and/or 14917^T^ 20174^T^; *L. argentorensis* DSM 16365^T^; *L. paraplantarum* DSM 10667^T^ and *L. pentosus* DSM 20314^T^.

Query genome	Reference genome	ANIb	ANIm	% dDDH (Formular 2*)	Model C.I. (%)	Distance	Prob. DDH >= 70%	G+C difference
1	2	98.92	99.27	98.3	97.5–98.8	0.0027	97.91	0.20
1	3	98.77	99.16	63.0	60.1–65.8	0.0465	61.85	0.74
1	4	94.78	95.66	32.0	29.6–34.5	0.1320	0.23	0.58
1	5	85.31	88.33	24.3	22.0–26.8	0.1793	0.01	2.03

*Notes*:

* Recommended formula (identities/HSP length), which is liberated of genome length and is thus prosperous against the use of draft genome.

**Table 3:** Genomic statistics of strain CRM56-2.

Attribute(s)	Value(s)
Scaffold	5
Scaffold	234,622
Scaffold	16
Scaffold	55,160
Scaffold length max	623,652
Scaffold length min	203
Scaffold length mean	16,537
Scaffold length median	317
Scaffold length standard deviation	66,461
Scaffold number A	942,533
Scaffold number T	937,131
Attribute(s)	Value(s)
Scaffold number C	733,213
Scaffold number G	760,421
Scaffold number N	313
Scaffold number bp	3,373,611
Scaffold number bp not N	3,373,298
Scaffold number sequence	204
Scaffold GC content overall	44.27
Contig L50	5
Contig N50	216,722
Contig L90	17
Contig N90	55,160
Contig length max	623,652
Contig length min	162
Contig length mean	15,689
Contig length median	316
Contig length standard deviation	63,630
Contig number bp	3,373,298
Contig number sequence	215
Number of gaps	11

**Table 4:** *In vitro* probiotic properties and cholesterol-lowering activities of strain CRM56-2.

Cholesterol-lowering activities and *in vitro* probiotic properties	

	*L. plantarum* CRM56-2
Cholesterol-lowering effects:	
• Bile salt hydrolase (BSH)	+
• Cholesterol assimilation (%)	75.94

- Simulated gastric phase (pH 3)	
• 0 h	2.4 × 10^7^
• 3 h	5.8 × 10^6^

- Simulated intestinal phase (pH 8.0, 0.3% bile salt)	
• 0 h	2.7 × 10^6^
• 3 h	7.7 × 10^6^

Adhesion ability (%)	0.40 ± 0.15

**Table 5:** Cholesterol-lowering genes (Deconjugation of bile salt and cholesterol assimilation).

Gene	Gene description	Length (bp)
For deconjugation of bile salt		
*bsh*	Choloylglycine hydrolase	975
For cholesterol assimilation abilities		
*ccpA*	Catabolite control protein A	978
*fba*	Class II fructose-1,6-bisphosphate aldolase	864
*glgP*	Glycogen phosphorylase	2,403
-	FMN-binding protein	369

*Note*: Data obtained from DFast annotation.

**Table 6:** Predicted proteins identified in the genome of *L. plantarum* CRM56-2 strain involved in acid and bile tolerance and adhesion/interaction.

Putative function	Genes	Predictive protein	Length (bp)
Adhesion or interaction with the host	*srtA*	Class A sortase	705
	*dltD*	D-Alanyl-lipoteichoic acid biosynthesis protein DltD	1,278
	*dltA*	D-Alanylation of LTA	1,527
	*glnH1*	Glutamine ABC transporter substrate- binding protein	837
	*lspA*	Lipoprotein signal peptidase	450
	*tuf*	Elongation factor Tu	1,188
	*mtsA*	Manganese ABC transporter substrate- binding protein	894
	*eno2*	Enolase 2	1,329
	*gapB*	Type I glyceraldehyde-3-phosphate dehydrogenase	1,002
	*groS*	Co-chaperonin GroES	285
	*groL*	Chaperonin GroEL	1,626
	*glnA*	Glutamine synthase	1,347
	*pgi*	Glucose-6-isomerase	1,353

Acid stress	*atpC*	ATP synthase subunit epsilon	429
	*atpD*	ATP synthase subunit beta	1,404
	*atpA*	ATP synthase subunit alpha	1,521
	*atpG*	ATP synthase subunit gamma	945
	*atpH*	ATP synthase subunit delta	546
	*atpF*	ATP synthase subunit B	516
	*atpB*	ATP synthase subunit A	714
	*atpE*	ATP synthase subunit C	213
	*recA*	Protein RecA (recombinase A)	1,143
	*relA*	GTP pyrophosphokinase	2,268
	*groS*	Co-chaperonin GroES	285
	*groL*	Chaperonin GroEL	1,626
	*htrA*	Serine protease	1,263
	*aspS*	Aspartate-tRNA ligase	1,797

Acid stress/Bile resistance	*gpmA1*	2,3-Bisphosphoglycerate dependent phosphoglycerate mutase 1	639
	*gpmA2*	2,3-Bisphosphoglycerate-dependent phosphoglycerate mutase 2	693
	*dnaK*	Chaperone protein DnaK	1,869
	*dnaJ*	Chaperone protein DnaJ	1,143
	*glmU*	Bifunctional UDP-N acetylglucosamine diphosphorylase/glucosamine phosphate	1,383
	*luxS*	S-Ribosylhomocysteine lyase	477
	*gadB*	Glutamate decarboxylase; GABA transporter	1,410
	*nha1*	Na^+^/H^+^ antiporter NhaC	1,425
	*nha2*	Na^+^/H^+^ antiporter NhaC	1,401
	*clpX*	ATP-dependent Clp protease ATP-binding subunit clpX	1,266

Bile resistance	*nagB*	Glucosamine-6-phosphate deaminase	714
	*pyrG*	CTP synthase	1,614
	*argS*	Arginine-tRNA ligase	1,689
	*rpsC*	30S Ribosomal protein S3	654
	*rpsE*	30S Ribosomal protein S5	501
	*rplD*	50S Ribosomal protein L4	624
	*rplE*	50S Ribosomal protein L5	543
	*rplF*	50S Ribosomal protein L6	537

*Note*: Data obtained DFast annotation.

**Table 7:** Pathogenicity prediction, prophage detection and antibiotic resistance genes (ARGs) analysis from PathogenFinder and ResFinder of CGE and PHASTER (Default program settings applied).

Attribute/Strain	*L. plantarum* CRM56-2	*L. plantarum* 299v
Probability of being a human pathogen	0.211	0.185
Input proteome coverage (%)	0.82	0.48
Matched pathogenic families	0	0
Matched not pathogenic families	26	15
Conclusion	Non-human pathogen	Non-human pathogen
No. of phage regions	6	4
ResFinder	No resistance	No resistance
