# Supplementary material for: Genomic Assessment of Potential Probiotic Lactiplantibacillus plantarum CRM56-2 Isolated from Fermented Tea Leaves
Source: Trop Life Sci Res. 2024 Jul 31;35(2):249–69. doi: 10.21315/tlsr2024.35.2.12 (PMC11371405; doi:10.21315/tlsr2024.35.2.12)
Supplement: Supplementary file 1 [file TLSR-35-2-249-Supplementary_Materials.pdf]

SUPPLEMENTARY MATERIAL

Genomic Assessment of Potential Probiotic *Lactiplantibacillus plantarum* CRM56-2 Isolated from Fermented Tea Leaves

[1,2]Engkarat Kingkaew, [3]Naoto Tanaka, [3]Yuh Shiwa, [4] Jaruwan Sitdhipol, [1]Rattanatda Nuhwa and [1]Somboon Tanasupawat*

[1]Department of Biochemistry and Microbiology, Faculty of Pharmaceutical Sciences, Chulalongkorn University, Bangkok 10330, Thailand
[2]Department of Biology, School of Science, King Mongkut's Institute of Technology Ladkrabang, Bangkok, 10520, Thailand
[3]Department of Molecular Microbiology, Faculty of Life Sciences, Tokyo University of Agriculture, 1-1-1 Sakuragaoka, Setagaya-ku, Tokyo 156-8502, Japan
[4]Biodiversity Research Centre, Research and Development Group for Bio-Industries, Thailand Institute of Scientific and Technological Research, Pathum Thani 12120, Thailand

***Corresponding author:** somboon.t@chula.ac.th

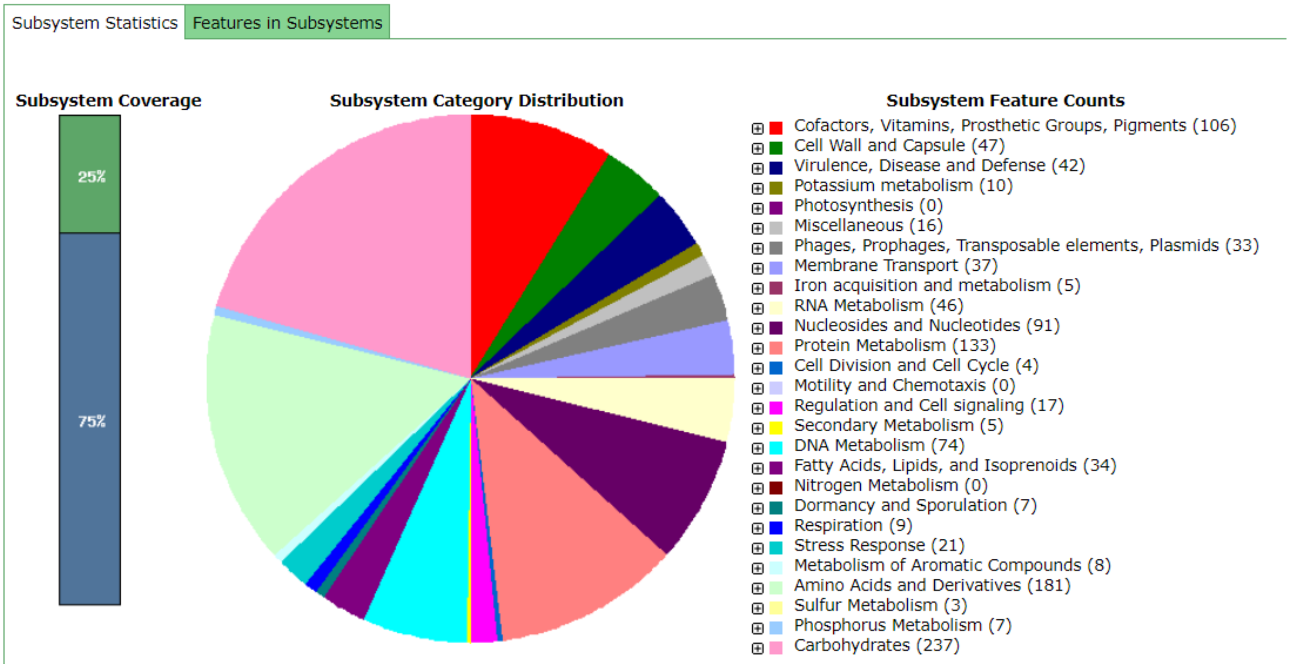

Fig. S1. An overview of the subsystem categories assigned to the genome of *L. plantarum* CRM56-2. The genomic sequence of the strain CRM56-2 was annotated using the Rapid Annotation System Technology (RAST) server. The pie chart demonstrates the count of each subsystem feature and the subsystem coverage.

Table S1. Proteolytic enzymes predicted in the genome of strain CRM56-2.

Category	Gene	Annotation/Substrate
**Proteinase**		
Cell wall-bound proteinase	*prtM*	Peptidylprolyl isomerase
**Peptidases**		
Aminopeptidase	*pepE*	Aminopeptidase
	pepN	Metallopeptidase
	pepC	Aminopeptidase C
	pepM	Methionine Aminopeptidase
	pcp	Pyroglutamyl-peptidase I
Dipeptidase	*pepD*	Dipeptidase
	pepA	M42 glutamyl aminopeptidase
	pepV	Dipeptidase PepV
Tripeptidase	*pepT*	Amino tripeptidase T
Endopeptidase	*pepF*	Oligoendopeptidase F
	pepO	Peptidase M13
Proline Peptidase	*pepR*	Proline peptidase
	pepX	Xaa-Pro dipeptidase
	pepQ	Xaa-Pro dipeptidase
	pepI	Proline aminopeptidase
	pepP	Xaa-Pro Aminopeptidase
**Transporters**		
Oligopeptide ABC transporter	*oppD*	Oligopeptide ABC transporter
	oppC	Oligopeptide ABC transporter
	oppF	Oligopeptide ABC transporter
	oppA	Oligopeptide ABC transporter
	oppB	Oligopeptide ABC transporter

Table S2. Predicted genes identified in the genome of CRM56-2 involved in transport and carbohydrates metabolism.

Predicted protein	Gene	Carbohydrate Type
**Transporters**		
PTS cellobiose transporter subunit IIA	*pts23A*	Cellobiose
PTS cellobiose transporter subunit IIA	*pts20A*	Cellobiose
PTS mannose transporter subunit IIC	-	Mannose
PTS mannose transporter subunit IIA	*pts10A*	Mannose
PTS mannose subunit IID	*pts9D*	Mannose
PTS glucose transporter subunit IIA	*pts17A*	Glucose
PTS glucose transporter subunit IIA	*pts32A*	Glucose
PTS sorbitol transporter subunit IIA	*pts38A*	Sorbitol
PTS sorbitol transporter subunit IIB	*pts38BC*	Sorbitol
PTS sorbitol transporter subunit IIC	*pts38C*	Sorbitol
PTS mannose/fructose/sorbose transporter subunit IIC	*pts9C*	Mannose/fructose/sorbose
PTS fructose transporter subunit IIB	-	Fructose
PTS fructose transporter subunit IIC	-	Fructose

PTS fructose transporter subunit IIC	*fruA*	Fructose
PTS fructose transporter subunit IIB	*pts10B*	Fructose
PTS fructose transporter subunit IIB	*pts11BC*	Fructose
PTS fructose transporter subunit IIA	*pts31A*	Fructose
PTS fructose transporter subunit IIBC	*pts31BC*	Fructose
PTS sugar transporter IIA	–	–
PTS sugar transporter IIA	*lacS*	–
PTS sugar transporter IIA	*pts11A*	–
PTS sugar transporter IIB	*pts15B*	–
PTS sugar transporter IIB	*pts20B*	–
PTS sugar transporter IIA	*pts21A*	–
PTS sugar transporter IIB	*pts23B*	–
PTS sugar transporter IIA	*rafp*	–
Phosphocarrier protein HPR	*ptsH*	–
Phosphoenolpyruvate--protein phosphotransferase	*ptsI*	–
**Enzymes**		
alpha-glucosidase	*agl*	Glucose, Galactose
Glucokinase	*glcK*	Sucrose
6-phospho-beta-glucosidase	*pbg1*	Glucose, Galactose, Chitobiose
6-phospho-beta-glucosidase	*pbg10*	Glucose, Galactose, Chitobiose
6-phospho-beta-glucosidase	*pbg3*	Glucose, Galactose, Chitobiose
6-phospho-beta-glucosidase	*pbg4*	Glucose, Galactose, Chitobiose
6-phospho-beta-glucosidase	*pbg5*	Glucose, Galactose, Chitobiose
6-phospho-beta-glucosidase	*pbg6*	Glucose, Galactose, Chitobiose
6-phospho-beta-glucosidase	*pbg7*	Glucose, Galactose, Chitobiose
6-phospho-beta-glucosidase	*pbg8*	Glucose, Galactose, Chitobiose
6-phospho-beta-glucosidase	*pbg9*	Glucose, Galactose, Chitobiose
mannose-6-phosphate isomerase	*pmi*	Mannose
glucose-6-phosphate isomerase	*pgi*	Sucrose
ATP-dependent-6-phosphofructokinase	*pfkA*	Fructose
galactose mutarotase	-	Galactose
galactose mutarotase	*galM2*	Galactose
galactose mutarotase	*galM3*	Galactose
LacI family transcriptional regulator	*scrR*	Sucrose
Sucrose-6-phosphate hydrolase	*scrB*	Sucrose
Phosphoglucomutase	*pgm*	Galactose, Sucrose
UDP-glucose 4-epimerase GalE	*galE1*	Galactose
UDP-glucose 4-epimerase GalE	*galE2*	Galactose

Table S3: Predicted proteins identified in the core genome of probiotic *L. plantarum* CRM56-2 involved in metabolic pathways of organic acids and vitamins biosynthesis.

Predicted Protein	Gene	Metabolites
Substrate-specific component FolT of folate ECF transporter	–	Folate
Methylenetetrahydrofolate--tRNA-(uracil(54)-C(5))-methyltransferase (FADH(2)-oxidizing) TrmFO	*trmFO*	Folate
Formate--tetrahydrofolate ligase	*fhs*	Folate
Dihydrofolate reductase	*dfrA*	Folate
Bifunctional folylpolyglutamate synthase/dihydrofolate synthase	*folC1*	Folate
Bifunctional folylpolyglutamate synthase/dihydrofolate synthase	*folC2*	Folate
Bifunctional methylenetetrahydrofolate dehydrogenase/methenyltetrahydrofolate cyclohydrolase	*folD*	Folate
Diacylglycerol kinase	-	Folate
5-formyltetrahydrofolate cyclo-ligase	*fthC*	Folate
Thiamine pyrophosphokinase	*tpk*	Thiamine
Thiamine-phosphate synthase	*thiE*	Thiamine
Riboflavin transporter	-	Riboflavin
Riboflavin biosynthesis protein RibC	*ribC1*	Riboflavin
Riboflavin biosynthesis protein	*ribC2*	Riboflavin
Riboflavin biosynthesis protein RibD	*ribD*	Riboflavin
Riboflavin kinase	-	Riboflavin
Glucose-6-phosphate isomerase	*pgi*	Lactate
Glucokinase	-	Lactate
6-phosphofructokinase	*pfkA*	Lactate
Glucose-6-phosphate dehydrogenase	*gpd*	Lactate
Ribulose-5-phosphate 3-epimerase	*rpe*	Lactate
Fructose-1,6-bisphosphate aldolase	*fba*	Lactate
Triose-phosphate isomerase	*tpiA*	Lactate
Glyceraldehyde-3-phosphate dehydrogenase	*gap*	Lactate
Phosphoglycerate kinase	*pgk*	Lactate
Phosphoglycerate mutase	*gpmA1*	Lactate
Phosphoglycerate mutase	*gpmA2*	Lactate
Enolase	*eno*	Lactate
Pyruvate kinase	*pyk*	Lactate
D-lactate dehydrogenase	*ldhD*	Lactate
L-lactate dehydrogenase	*ldh*	Lactate
Phosphoketolase	*xfp*	Acetate
Phosphate acetyltransferase	*pta*	Acetate
Acetate kinase	*ackA*	Acetate

Hydroxymethylglutaryl-CoA synthase	*mvaS*	Butyrate
Formate C-acetyltransferase	*pflB*	Butyrate
Succinate-semialdehyde	*gabD*	Butyrate
Dehydrogenase/glutarate-semialdehyde dehydrogenase		Butyrate
Acetolactate decarboxylase	*aldB*	Butyrate
Acetolactate synthase	*als*	Butyrate
Fumarate reductase flavoprotein subunit		Butyrate
Aldehyde-Alcohol dehydrogenase	*adhE*	Butyrate
Glutamate decarboxylase	*gadB*	Butyrate
D-lactate dehydrogenase	*ldhD*	Butyrate
L-lactate dehydrogenase	*ldh*	Butyrate
Phosphate acetyltransferase	*pta*	Butyrate
Acetate kinase	*ack1*	Butyrate
Acetate kinase	*ack2*	Butyrate
Acetate kinase	*ackA*	Butyrate
3-oxoacyl-ACP synthase III	*fabh1*	Butyrate
Biotin carboxyl carrier protein	*accb1*	Butyrate
Biotin carboxylase subunit	*accC1*	Butyrate
Carboxyl transferase subunit β	*accD1*	Butyrate
Carboxyl transferase subunit α	*accA2*	Butyrate
Phosphate acyltransferase	*plsX*	Butyrate
Acyl carrier protein (ACP)	*acpA1*	Butyrate
3-hydroxyacyl-[acyl-carrier-protein] dehydratase	*fabZ*	Butyrate
3-oxoacyl-[acyl-carrier-protein] synthase 3 protein 2	*fabH2*	Butyrate
Acyl carrier protein	*acpA2*	Butyrate
malonyl CoA-acyl carrier protein transacylase	*fabD*	Butyrate
3-oxoacyl-ACP reductase	*fabG1*	Butyrate
3-oxoacyl-ACP synthase II	*fabF*	Butyrate
Biotin carboxyl carrier protein	*accB2*	Butyrate
(3R)-hydroxymyristoyl-ACP dehydratase	*fabZ2*	Butyrate
Biotin carboxylase subunit	*accC2*	Butyrate
Carboxyl transferase subunit β	*accD2*	Butyrate
Carboxyl transferase subunit α	*accA2*	Butyrate
Enoyl-ACP reductase	*fabI*	Butyrate
Holo-ACP synthase (ACPS)	*acps*	Butyrate
Acyl-ACP thioesterase	-	Butyrate
Biotin carboxyl carrier protein	*accB3*	Butyrate
3-oxoacyl-ACP reductase	*fabG*	Butyrate
Putative enoyl-ACP reductase (FabI)	*fabI*	Butyrate

Table S4: dbCAN

Gene ID	EC#	HMMER	dbCAN_sub	DIAMOND	Signalp	#ofTools
JAEMUU010000012.1_43	N	CBM34(6-127)+GH13_20(176-474)	N	CBM34+GH13_20	N	2
JAEMUU010000002.1_258	N	CBM48(27-106)+GH13_9(174-473)	N	CBM48+GH13_9	N	2
JAEMUU010000002.1_159	N	GH1(2-476)	N	GH1	N	2
JAEMUU010000030.1_32	N	GH1(2-481)	N	GH1	N	2
JAEMUU010000009.1_146	N	GH1(3-479)	N	GH1	N	2
JAEMUU010000001.1_69	N	GH1(4-457)	N	GH1	N	2
JAEMUU010000005.1_83	N	GH1(6-474)	N	GH1	N	2
JAEMUU010000012.1_25	N	GH1(6-476)	N	GH1	N	2
JAEMUU010000012.1_26	N	GH1(7-476)	N	GH1	N	2
JAEMUU010000002.1_147	N	GH1(8-482)	N	GH1	N	2
JAEMUU010000002.1_160	N	GH1(9-483)	N	GH1	N	2
JAEMUU010000011.1_54	N	GH126(46-365)	N	GH126	N	2
JAEMUU010000002.1_385	N	GH13(25-314)	N	GH13	N	2
JAEMUU010000002.1_452	N	GH13_29(24-370)	N	GH13_29	N	2
JAEMUU010000009.1_73	N	GH13_31(28-378)	N	GH13_31	N	2
JAEMUU010000002.1_397	N	GH13_31(28-379)	N	GH13_31	N	2
JAEMUU010000002.1_380	N	GH13_31(28-384)	N	GH13	N	2
JAEMUU010000013.1_89	N	GH13_31(29-378)	N	GH13_31	N	2
JAEMUU010000002.1_394	N	GH13_31(30-378)	N	GH13_31	N	2
JAEMUU010000002.1_263	N	GH13_39(188-496)	N	GH13_39	N	2
JAEMUU010000002.1_158	N	GH170(1-344)	N	GH170	N	2
JAEMUU010000012.1_34	N	GH170(3-360)	N	GH170	N	2
JAEMUU010000002.1_146	N	GH170(5-361)	N	GH170	N	2
JAEMUU010000002.1_120	N	GH2(19-622)	N	GH2	N	2
JAEMUU010000009.1_182	N	GH25(232-408)	N	GH25	N	2
JAEMUU010000014.1_178	N	GH25(30-195)	N	GH25	N	2

JAEMUU010000001.1_236	N	GH25(33-202)	N	GH25	N	2
JAEMUU010000013.1_146	N	GH25(35-210)	N	CBM50+GH25	N	2
JAEMUU010000017.1_18	N	GH25(37-221)	N	CBM50+GH25	N	2
JAEMUU010000030.1_4	N	GH25(37-221)	N	CBM50+GH25	N	2
JAEMUU010000018.1_46	N	GH25(60-225)	N	GH25	N	2
JAEMUU010000002.1_166	N	GH31(230-671)	N	GH31	N	2
JAEMUU010000002.1_392	N	GH32(37-339)	N	GH32	N	2
JAEMUU010000002.1_122	N	GH36(18-718)	N	GH36	N	2
JAEMUU010000009.1_63	N	GH38(3-276)	N	GH38	N	2
JAEMUU010000002.1_107	N	GH42(15-394)	N	GH42	N	2
JAEMUU010000002.1_163	N	GH65(306-670)	N	GH65	N	2
JAEMUU010000014.1_147	N	GH65(318-688)	N	GH65	N	2
JAEMUU010000002.1_387	N	GH65(322-693)	N	GH65	N	2
JAEMUU010000002.1_267	N	GH65(427-836)	N	GH65	N	2
JAEMUU010000010.1_2	N	GH70(482-1270)	N	GH70	N	2
JAEMUU010000009.1_131	N	GH73(11-81)	N	N	N	1
JAEMUU010000012.1_133	N	GH73(255-385)	N	GH73	N	2
JAEMUU010000018.1_63	N	GH73(71-206)	N	GH73	N	2
JAEMUU010000002.1_109	N	GH78(159-615)	N	GH78	N	2
JAEMUU010000021.1_2	N	GH78(66-519)	N	GH78	N	2
JAEMUU010000002.1_111	N	GH78(71-524)	N	GH78	N	2
JAEMUU010000002.1_388	N	GH85(109-451)+CBM32(813-938)	N	CBM32+GH85	N	2

Table S5: Phage summary.

Strain: *L. plantarum* CRM56-2
Total: 6 prophage regions have been identified, of which 2 regions are intact, 3 regions are incomplete, and 1 regions are questionable.

Region	Region length	Completeness	Score	# Total Proteins	Region position	Most common phage
1	10.5Kb	incomplete	60	21	7214-17787	PHAGE_Bacill_vB_BtS_BMBtp14_NC_048640(2)

2	44Kb	intact	110	61	142119-186170	PHAGE_Oenoco_phiS13_NC_023560(15)
3	23.3Kb	incomplete	60	26	186252-209604	PHAGE_Lactob_Lrm1_NC_011104(3)
4	29.5Kb	intact	100	41	333-29910	PHAGE_Lactob_Sha1_NC_019489(18)
5	5.4Kb	incomplete	20	7	75229-80668	PHAGE_Bacill_vB_BtS_BMBtp14_NC_048640(2)
6	18.8Kb	questionable	70	29	1-18800	PHAGE_Lactob_Sha1_NC_019489(18)

Strain: *L. plantarum* 299v
Total: 4 prophage regions have been identified, of which 2 regions are intact, 1 regions are incomplete, and 1 regions are questionable.

Region	Region length	Completeness	Score	# Total Proteins	Region position	Most common phage
1	7.1Kb	incomplete	40	13	19349-26470	PHAGE_Mycoba_Adler_NC_023591(1)
2	39.9Kb	intact	150	54	140793-180732	PHAGE_Lactob_Sha1_NC_019489(27)
3	39.5Kb	intact	130	55	44-39629	PHAGE_Lister_B025_NC_009812(9)
4	14.2Kb	questionable	90	23	105-14324	PHAGE_Entero_EFRM31_NC_015270(2)

Prophages identified in the genome of *L. plantarum* CRM56-2
Highlight pink = phage DNA; purple = bacterial DNA

#	CDS Position	BLAST Hit	E-Value
		**Region 1, incomplete, total 21 CDS**	
1	7214..7579	PROPHAGE_Xantho_33913: IS1480 transposase; PP_01179; phage(gi21231089)	1.19E-30
2	7616..8596	hypothetical; PP_01180	0
3	8645..9838	hypothetical; PP_01181	0
4	9843..10295	PHAGE_Strept_Samisti12_NC_042012: holin; PP_01182; phage(gi100034)	2.87E-22

5	complement(10380..10511)	PHAGE_Paenib_BN12_NC_048688: hypothetical protein; PP_01183; phage(gi100028)	7.91E-06
6	complement(10597..11055)	PHAGE_Salmon_118970_sal4_NC_030919: hypothetical protein; PP_01184; phage(gi100002)	1.28E-07
7	complement(11076..11339)	hypothetical; PP_01185	0
8	11420..11719	PROPHAGE_Shewan_MR-1: ISSod6, transposase; PP_01186; phage(gi24374783)	1.39E-05
9	11968..12219	PROPHAGE_Xantho_33913: IS1480 transposase; PP_01187; phage(gi21231089)	5.37E-22
10	complement(12314..12388)	hypothetical; PP_01188	0
11	12411..12494	hypothetical; PP_01189	0
12	12554..13324	hypothetical; PP_01190	0
13	13336..14055	hypothetical; PP_01191	0
14	14123..14815	hypothetical; PP_01192	0
15	complement(14943..15224)	PHAGE_Faecal_FP_Lagaffe_NC_047911: putative phosphoesterase; PP_01193; phage(gi100037)	3.81E-11
16	complement(15238..15489)	PROPHAGE_Xantho_33913: IS1480 transposase; PP_01194; phage(gi21231089)	9.04E-24
17	complement(15507..15698)	PROPHAGE_Xantho_33913: IS1480 transposase; PP_01195; phage(gi21231089)	1.94E-17
18	complement(15766..15921)	hypothetical; PP_01196	0
19	complement(15970..16212)	hypothetical; PP_01197	0
20	complement(16377..17003)	PHAGE_Bacill_vB_BtS_BMBtp14_NC_048640: methyltransferase type 11; PP_01198; phage(gi100001)	1.23E-46
21	complement(17329..17787)	PHAGE_Bacill_vB_BtS_BMBtp14_NC_048640: hypothetical protein; PP_01199; phage(gi100002)	1.60E-13
**Region 2, intact, total 63 CDS**			
1	complement(142119..143888)	PHAGE_Bacill_SPbeta_NC_001884: ABC transporter; PP_02144; phage(gi9630145)	6.25E-31

2	complement(144008..145756)	PHAGE_Bacill_SPbeta_NC_001884: ABC transporter; PP_02145; phage(gi9630145)	5.51E-18
3	complement(145862..145945)	hypothetical; PP_02146	0
4	complement(145962..146354)	PHAGE_Staphy_StB20_like_NC_028821: hypothetical protein; PP_02147; phage(gi971746471)	3.06E-06
5	146562..147428	PHAGE_Bacill_BCP8_2_NC_027355: putative polysaccharide deacetylase; PP_02148; phage(gi849252969)	1.72E-06
6	147566..147676	hypothetical; PP_02149	0
7	147997..148842	hypothetical; PP_02150	0
8	complement(149048..149260)	PHAGE_Lactob_phig1e_NC_004305: lysin; PP_02151; phage(gi254733530)	1.89E-33
9	complement(149939..150280)	PHAGE_Staphy_DW2_NC_024391: holin; PP_02152; phage(gi658608090)	3.58E-07
10	complement(150303..150599)	PHAGE_Lactob_Nyseid_NC_047925: hypothetical protein; PP_02153; phage(gi100097)	3.04E-51
11	complement(150600..151766)	PHAGE_Lactob_phig1e_NC_004305: lysin; PP_02154; phage(gi254733530)	6.16E-70
12	complement(151894..152328)	hypothetical; PP_02155	0
13	complement(152331..152780)	hypothetical; PP_02156	0
14	complement(152802..158054)	PHAGE_Lactob_Lj965_NC_005355: hypothetical protein; PP_02157; phage(gi41179262)	2.42E-164
15	complement(158069..158431)	PHAGE_Lactob_Lj965_NC_005355: hypothetical protein; PP_02158; phage(gi41179261)	8.54E-43
16	complement(158445..164276)	PHAGE_Lactob_Lj965_NC_005355: putative putative minor tail protein; PP_02159; phage(gi41179260)	0
17	complement(164292..164636)	PHAGE_Lactob_Lj965_NC_005355: hypothetical protein; PP_02160; phage(gi41179259)	6.34E-32

18	complement(164663..165061)	PHAGE_Lactob_Lj965_NC_005355: hypothetical protein; PP_02161; phage(gi41179250)	4.48E-46
19	complement(165161..165637)	PHAGE_Lactob_Lj965_NC_005355: putative major tail protein; PP_02162; phage(gi41179249)	2.11E-62
20	complement(165646..165996)	PHAGE_Lactob_Lj965_NC_005355: hypothetical protein; PP_02163; phage(gi41179248)	1.02E-39
21	complement(166011..166562)	PHAGE_Lactob_Lj965_NC_005355: hypothetical protein; PP_02164; phage(gi41179258)	1.38E-70
22	complement(166564..166797)	PHAGE_Lactob_Lj965_NC_005355: hypothetical protein; PP_02165; phage(gi41179247)	9.84E-30
23	complement(166911..167243)	PHAGE_Lactob_Lj965_NC_005355: hypothetical protein; PP_02166; phage(gi41179246)	3.14E-16
24	complement(167255..167431)	hypothetical; PP_02167	0
25	complement(167444..168466)	PHAGE_Lactob_Lj965_NC_005355: putative major head protein; PP_02168; phage(gi41179245)	5.43E-120
26	complement(168486..168833)	PHAGE_Oenoco_phiS13_NC_023560: prophage protein; PP_02169; phage(gi589286250)	1.86E-41
27	complement(168848..169525)	PHAGE_Lactob_Lj965_NC_005355: putative scaffold protein; PP_02170; phage(gi41179243)	5.13E-26
28	complement(169697..169903)	hypothetical; PP_02171	0
29	complement(169955..170155)	hypothetical; PP_02172	0
30	complement(170208..171893)	PHAGE_Lactob_Lj965_NC_005355: putative minor head protein; PP_02173; phage(gi41179242)	9.13E-113
31	complement(171910..172032)	hypothetical; PP_02174	0
32	complement(172040..172225)	hypothetical; PP_02175	0

33	complement(172266..173774)	PHAGE_Lactob_Lj965_NC_005355: putative portal protein; PP_02176; phage(gi41179240)	1.04E-150
34	complement(173786..174985)	PHAGE_Lactoc_PLgT_1_NC_031016: hypothetical protein; PP_02177; phage(gi100030)	3.36E-165
35	complement(175014..175541)	PHAGE_Lactob_phig1e_NC_004305: putative terminase small subunit; PP_02178; phage(gi23455798)	5.46E-63
36	complement(175576..175692)	hypothetical; PP_02179	0
37	complement(175719..175898)	PHAGE_Lactob_Sha1_NC_019489: HNH nuclease; PP_02180; phage(gi418489799)	1.53E-11
38	complement(176008..176829)	hypothetical; PP_02181	0
39	complement(176957..177106)	hypothetical; PP_02182	0
40	complement(177016..177087)	tRNA	0
41	complement(177113..177229)	hypothetical; PP_02183	0
42	complement(177157..177229)	tRNA	0
43	complement(177352..177813)	PHAGE_Lactob_LBR48_NC_027990: putative transcription regulator; PP_02184; phage(gi937456737)	1.33E-54
44	complement(177891..177980)	hypothetical; PP_02185	0
45	complement(178024..178392)	PHAGE_Lister_A118_NC_003216: gp63; PP_02186; phage(gi16798850)	5.87E-35
46	complement(178401..178904)	PHAGE_Lactob_phig1e_NC_004305: hypothetical protein; PP_02187; phage(gi23455787)	8.86E-70
47	complement(178916..179122)	hypothetical; PP_02188	0

48	complement(179200..180108)	PHAGE_Lactob_phig1e_NC_004305: hypothetical protein; PP_02189; phage(gi254854754)	3.18E-38
49	complement(180187..180939)	PHAGE_Lactob_iLp84_NC_028783: hypothetical protein; PP_02190; phage(gi971743105)	1.45E-93
50	complement(180971..181858)	PHAGE_Lactob_LF1_NC_019486: RecT protein; PP_02191; phage(gi418489428)	6.23E-85
51	complement(181861..182292)	hypothetical; PP_02192	0
52	complement(182374..182511)	hypothetical; PP_02193	0
53	complement(182516..182599)	hypothetical; PP_02194	0
54	complement(182612..183124)	PHAGE_Lactob_LBR48_NC_027990: putative transcription regulator; PP_02195; phage(gi937456713)	5.60E-41
55	complement(183192..183497)	hypothetical; PP_02196	0
56	complement(183509..183679)	hypothetical; PP_02197	0
57	complement(183704..183829)	hypothetical; PP_02198	0
58	complement(183838..184038)	PHAGE_Thermo_THSA_485A_NC_018264: transcriptional regulator, XRE family; PP_02199; phage(gi397912660)	1.40E-07
59	184185..184421	PHAGE_Brevib_Osiris_NC_028969: hypothetical protein; PP_02200; phage(gi971761833)	1.53E-13
60	184459..184773	hypothetical; PP_02201	0
61	complement(184830..185177)	PHAGE_Lactob_LBR48_NC_027990: putative transcription regulator; PP_02202; phage(gi937456709)	4.00E-08
62	185230..185733	PHAGE_Thermu_OH2_NC_021784: immunity repressor protein (phage-like protein); PP_02203; phage(gi526118362)	1.83E-32

63	185748..186170	PHAGE_Bacill_BM5_NC_029069: hypothetical protein; PP_02204; phage(gi985757868)	2.22E-16
**Region 3, incomplete, total 31 CDS**			
1	186252..186263	attL	0
2	193605..194726	PHAGE_Lactob_Lrm1_NC_011104: integrase; PP_02217; phage(gi195661224)	1.79E-48
3	194740..194913	hypothetical; PP_02218	0
4	complement(194955..195027)	tRNA	0
5	complement(195077..195283)	hypothetical; PP_02219	0
6	195411..195557	hypothetical; PP_02220	0
7	195418..195490	tRNA	0
8	complement(195701..196006)	hypothetical; PP_02221	0
9	complement(196088..196459)	PHAGE_Lactob_Lrm1_NC_011104: hypothetical protein; PP_02222; phage(gi195661249)	2.85E-06
10	complement(196616..196885)	PHAGE_Entero_IME_EFm1_NC_024356: head-tail joining protein; PP_02223; phage(gi658310466)	2.59E-08
11	196954..197025	tRNA	0
12	complement(197083..197193)	hypothetical; PP_02224	0
13	complement(197295..198800)	PHAGE_Staphy_PVL_NC_002321: capsid protein; PP_02225; phage(gi9635171)	2.50E-44
14	complement(198833..199933)	PHAGE_Lactob_Lrm1_NC_011104: portal protein; PP_02226; phage(gi195661204)	2.17E-49
15	complement(199934..200065)	hypothetical; PP_02227	0
16	complement(200088..201770)	PHAGE_Strept_315.2_NC_004585: hypothetical protein; PP_02228; phage(gi28876230)	2.08E-132

17	complement(201788..202261)	PHAGE_Entero_vB_EfaS_AL2_NC_042127: hypothetical protein; PP_02229; phage(gi100060)	3.56E-15
18	202398..202409	attR	0
19	202404..202643	hypothetical; PP_02230	0
20	complement(202973..203350)	PHAGE_Staphy_Pvl108_NC_008689: phage endonuclease; PP_02231; phage(gi119443688)	6.32E-23
21	complement(203355..203558)	hypothetical; PP_02232	0
22	complement(203680..203859)	hypothetical; PP_02233	0
23	complement(203871..204227)	PHAGE_Staphy_PT1028_NC_007045: ORF013; PP_02234; phage(gi66395177)	7.14E-12
24	204279..204434	hypothetical; PP_02235	0
25	complement(204489..205796)	PHAGE_Microb_Zeta1847_NC_047992: hypothetical protein; PP_02236; phage(gi100038)	6.10E-36
26	complement(206063..206788)	PHAGE_Lactoc_bIL310_NC_002669: hypothetical protein; PP_02237; phage(gi13095885)	3.03E-21
27	complement(206863..207081)	hypothetical; PP_02238	0
28	complement(207214..207321)	hypothetical; PP_02239	0
29	complement(207363..207539)	hypothetical; PP_02240	0
30	207692..208372	PHAGE_Lactob_phiAQ113_NC_019782: putative transcriptional regulator; PP_02241; phage(gi446730276)	6.53E-18
31	208447..209604	PROPHAGE_Oceano_HTE831: integrase; PP_02242; phage(gi23097608)	2.19E-74
**Region 4, incomplete, total 45 CDS**			

1	complement(333..1547)	PHAGE_Lactob_Sha1_NC_019489: HK97 family phage major capsid protein; PP_02743; phage(gi418489806)	0
2	complement(1565..2251)	PHAGE_Lactob_Sha1_NC_019489: protease subunit of ATP-dependent Clp protease; PP_02744; phage(gi418489805)	1.13E-149
3	complement(2301..3494)	PHAGE_Lactob_Sha1_NC_019489: phage portal protein; PP_02745; phage(gi418489804)	0
4	2798..2813	attL	0
5	complement(3497..3625)	PHAGE_Lactob_Sha1_NC_019489: phage head-tail joining protein; PP_02746; phage(gi418489803)	1.23E-20
6	complement(3681..5579)	PHAGE_Lactob_Sha1_NC_019489: phage terminase-like protein large subunit; PP_02747; phage(gi418489802)	0
7	complement(5582..6040)	PHAGE_Lactob_Sha1_NC_019489: P27 family phage terminase small subunit; PP_02748; phage(gi418489801)	5.45E-107
8	complement(6239..6706)	PHAGE_Lactob_Sha1_NC_019489: restriction endonuclease; PP_02749; phage(gi418489800)	1.84E-106
9	complement(6751..7197)	PHAGE_Lactob_A2_NC_004112: hypothetical protein; PP_02750; phage(gi22296581)	5.81E-09
10	complement(7511..7942)	PHAGE_Lactob_Sha1_NC_019489: phage transcriptional activator RinA; PP_02751; phage(gi418489798)	9.80E-101
11	complement(8090..8419)	PHAGE_Lactob_A2_NC_004112: hypothetical protein; PP_02752; phage(gi22296564)	1.07E-36
12	complement(8433..8651)	PHAGE_Lactob_Lrm1_NC_011104: hypothetical protein; PP_02753; phage(gi195661245)	4.22E-30
13	complement(8813..9007)	hypothetical; PP_02754	0
14	complement(9161..9268)	PHAGE_Lactob_Sha1_NC_019489: hypothetical protein; PP_02755; phage(gi418489790)	3.04E-09
15	complement(9281..9469)	PHAGE_Lactob_LBR48_NC_027990: hypothetical protein; PP_02756; phage(gi937456731)	9.68E-38
16	complement(9483..9851)	PHAGE_Lister_A118_NC_003216: gp63; PP_02757; phage(gi16798850)	4.07E-34

17	complement(9860..10360)	PHAGE_Lactob_phig1e_NC_004305: hypothetical protein; PP_02758; phage(gi23455787)	2.61E-69
18	complement(10496..11233)	PHAGE_Lactob_Sha1_NC_019489: DNA replication protein; PP_02759; phage(gi418489789)	1.22E-180
19	complement(11281..12015)	PHAGE_Lactob_CL1_NC_028888: putative replication protein; PP_02760; phage(gi971752673)	8.42E-47
20	complement(12039..12179)	hypothetical; PP_02761	0
21	complement(12250..12942)	PHAGE_Lactob_Sha1_NC_019489: hypothetical protein; PP_02762; phage(gi418489846)	1.79E-168
22	complement(12989..13648)	PHAGE_Lactob_Sha1_NC_019489: hypothetical protein; PP_02763; phage(gi418489845)	1.00E-152
23	complement(13650..14312)	PHAGE_Lactob_Sha1_NC_019489: phage nucleotide-binding protein; PP_02764; phage(gi418489844)	1.73E-160
24	complement(14313..15137)	PHAGE_Lactob_CL1_NC_028888: hypothetical protein; PP_02765; phage(gi971752658)	6.07E-48
25	complement(15173..15319)	PHAGE_Lactob_Sha1_NC_019489: hypothetical protein; PP_02766; phage(gi418489842)	3.52E-26
26	complement(15322..15492)	PHAGE_Lactob_Sha1_NC_019489: hypothetical protein; PP_02767; phage(gi418489841)	1.39E-18
27	complement(15498..15740)	PHAGE_Lactob_Sha1_NC_019489: hypothetical protein; PP_02768; phage(gi418489840)	4.90E-40
28	complement(15788..15949)	PHAGE_Lactob_Sha1_NC_019489: hypothetical protein; PP_02769; phage(gi418489839)	4.14E-29
29	complement(16092..16352)	hypothetical; PP_02770	0
30	complement(16372..16575)	hypothetical; PP_02771	0
31	complement(16599..16757)	PHAGE_Lactob_Lb_NC_047983: putative transcriptional regulator; PP_02772; phage(gi100045)	1.25E-06
32	complement(16771..16980)	PHAGE_Lactob_phiadh_NC_000896: hypothetical protein; PP_02773; phage(gi9633008)	5.89E-06

33	complement(16992..17699)	PHAGE_Lactob_Sha1_NC_019489: phage-related antirepressor; PP_02774; phage(gi418489833)	3.65E-36
34	complement(17725..17922)	PHAGE_Strept_phiARI0746_NC_031907: hypothetical protein; PP_02775; phage(gi100038)	1.01E-06
35	18176..18508	PHAGE_Lactob_PLE2_NC_031036: hypothetical protein; PP_02776; phage(gi100029)	2.85E-37
36	18561..18908	PHAGE_Lactob_PLE2_NC_031036: hypothetical protein; PP_02777; phage(gi100028)	1.59E-32
37	18930..19847	hypothetical; PP_02778	0
38	19850..19969	hypothetical; PP_02779	0
39	20004..20089	tRNA	0
40	20261..21442	PHAGE_Lactob_LBR48_NC_027990: putative integrase; PP_02780; phage(gi937456703)	4.66E-79
41	complement(21564..21636)	tRNA	0
42	complement(21684..23018)	PHAGE_Lactob_phiadh_NC_000896: putative DEAH-family helicase; PP_02781; phage(gi9633020)	4.20E-11
43	complement(23031..24038)	hypothetical; PP_02782	0
44	complement(24225..24425)	PHAGE_Vibrio_1.026.O._10N.222.49.C7_NC_049430: portal protein; PP_02783; phage(gi100069)	1.26E-11
45	29910..29925	attR	0
**Region 5, incomplete, total 7 CDS**			
1	complement(75229..76158)	PHAGE_Lactob_LJ_NC_048680: hypothetical protein; PP_03314; phage(gi100060)	6.94E-53
2	complement(76759..77016)	hypothetical; PP_03315	0
3	77450..77863	PHAGE_Bacill_vB_BtS_BMBtp14_NC_048640: hypothetical protein; PP_03316; phage(gi100002)	2.73E-07
4	77923..78339	PHAGE_Bacill_vB_BtS_BMBtp14_NC_048640: methyltransferase type 11; PP_03317; phage(gi100001)	1.25E-19
5	78471..78728	PHAGE_Paenib_Leyra_NC_048692: hypothetical protein; PP_03318; phage(gi100029)	3.57E-16

6	complement(79123..79677)	PHAGE_Lactob_Lv_1_NC_011801: hypothetical protein; PP_03319; phage(gi219563221)	1.80E-06
7	complement(79742..80668)	PHAGE_Clostr_phiCDHM19_NC_028996: putative tail tape measure protein (partial); PP_03320; phage(gi971763886)	4.26E-05
		**Region 6, incomplete, total 30 CDS**	
1	complement(1..207)	PHAGE_Lactob_Sha1_NC_019489: phage protein DNA packaging protein; PP_03396; phage(gi418489807)	2.03E-35
2	complement(280..1512)	PHAGE_Lactob_Sha1_NC_019489: HK97 family phage major capsid protein; PP_03397; phage(gi418489806)	0
3	complement(1527..2198)	PHAGE_Lactob_Sha1_NC_019489: protease subunit of ATP-dependent Clp protease; PP_03398; phage(gi418489805)	1.56E-148
4	complement(2248..3441)	PHAGE_Lactob_Sha1_NC_019489: phage portal protein; PP_03399; phage(gi418489804)	0
5	complement(3444..3572)	PHAGE_Lactob_Sha1_NC_019489: phage head-tail joining protein; PP_03400; phage(gi418489803)	1.23E-20
6	complement(3628..5526)	PHAGE_Lactob_Sha1_NC_019489: phage terminase-like protein large subunit; PP_03401; phage(gi418489802)	0
7	complement(5536..5991)	PHAGE_Lactob_Sha1_NC_019489: P27 family phage terminase small subunit; PP_03402; phage(gi418489801)	3.68E-104
8	complement(6182..6448)	hypothetical; PP_03403	0
9	complement(6457..6726)	hypothetical; PP_03404	0
10	complement(6732..7202)	PHAGE_Lactob_Sha1_NC_019489: restriction endonuclease; PP_03405; phage(gi418489800)	7.96E-105
11	complement(7213..7383)	PHAGE_Lactob_Sha1_NC_019489: HNH nuclease; PP_03406; phage(gi418489799)	1.38E-32
12	complement(7543..8256)	hypothetical; PP_03407	0
13	complement(8542..8754)	hypothetical; PP_03408	0
14	complement(9025..9453)	PHAGE_Lactob_Sha1_NC_019489: phage transcriptional activator RinA; PP_03409; phage(gi418489798)	1.58E-82

15	complement(9728..10036)	PHAGE_Lactob_Sha1_NC_019489: hypothetical protein; PP_03410; phage(gi418489790)	1.31E-64
16	complement(10040..10894)	PHAGE_Lactob_LJ_NC_048680: hypothetical protein; PP_03411; phage(gi100007)	1.38E-49
17	complement(10918..11610)	PHAGE_Lactob_Sha1_NC_019489: hypothetical protein; PP_03412; phage(gi418489846)	8.45E-154
18	complement(11625..12683)	PHAGE_Lactob_CL1_NC_028888: single-stranded DNA-binding protein; PP_03413; phage(gi971752655)	3.07E-59
19	complement(12709..13569)	PHAGE_Lactob_CL1_NC_028888: hypothetical protein; PP_03414; phage(gi971752658)	5.52E-48
20	complement(13917..14117)	PHAGE_Lactob_Sha1_NC_019489: hypothetical protein; PP_03415; phage(gi418489840)	9.56E-37
21	complement(14120..14368)	PHAGE_Lactob_Sha1_NC_019489: hypothetical protein; PP_03416; phage(gi418489839)	6.87E-47
22	complement(14458..14787)	PHAGE_Lactob_Sha1_NC_019489: hypothetical protein; PP_03417; phage(gi418489836)	5.79E-65
23	14880..15089	hypothetical; PP_03418	0
24	complement(15260..15469)	PHAGE_Lactob_phiadh_NC_000896: hypothetical protein; PP_03419; phage(gi9633008)	2.60E-05
25	complement(15481..16188)	PHAGE_Lactob_Sha1_NC_019489: phage-related antirepressor; PP_03420; phage(gi418489833)	5.09E-38
26	complement(16204..16452)	PHAGE_Lactob_BH1_NC_048737: hypothetical protein; PP_03421; phage(gi100029)	1.25E-29
27	16612..17295	PHAGE_Lactob_Sha1_NC_019489: bifunctional S24 family peptidase/transcriptional regulator; PP_03422; phage(gi418489831)	8.67E-33
28	17409..18299	hypothetical; PP_03423	0
29	18456..18546	tRNA	0
30	18600..18800	PHAGE_Lactob_Sha1_NC_019489: hypothetical protein; PP_03424; phage(gi418489829)	8.02E-36

Prophages identified in the genome of *L. plantarum* 299v

Highlight pink= phage DNA; purple=bacterial DNA

#	CDS Position	BLAST Hit	E-Value
		**Region 1, incomplete, total 13 CDS**	
1	complement(19349..20215)	PHAGE_Entero_EF62phi_NC_017732: chromosome partitioning ATPase; PP_01101; phage(gi384519759)	2.37E-73
2	complement(20511..20588)	hypothetical; PP_01102	0
3	complement(20750..20872)	hypothetical; PP_01103	0
4	21322..21510	hypothetical; PP_01104	0
5	complement(21904..22437)	PROPHAGE_Xantho_33913: IS1480 transposase; PP_01105; phage(gi21231089)	2.02E-62
6	22594..22815	PHAGE_Staphy_phiPV83_NC_002486: transposase; PP_01106; phage(gi9635740)	1.72E-06
7	22855..23523	PHAGE_Staphy_phiPV83_NC_002486: transposase; PP_01107; phage(gi9635740)	3.41E-38
8	complement(23572..23871)	hypothetical; PP_01108	0
9	complement(23873..24733)	PHAGE_Entero_EF62phi_NC_017732: chromosome partitioning ATPase; PP_01109; phage(gi384519759)	2.06E-56
10	25110..25316	hypothetical; PP_01110	0
11	complement(25323..25442)	hypothetical; PP_01111	0
12	25742..26020	hypothetical; PP_01112	0
13	complement(26222..26470)	PHAGE_Lactob_SA_C12_NC_047760: hypothetical protein; PP_01113; phage(gi100004)	4.52E-08
		**Region 2, intact, total 57 CDS**	
1	140793..140806	attL	0
2	complement(140889..142052)	PHAGE_Lactob_LF1_NC_019486: phage integrase; PP_01387; phage(gi418489411)	1.43E-78
3	complement(142220..142534)	hypothetical; PP_01388	0

4	complement(142718..142975)	hypothetical; PP_01389	0
5	complement(143101..143277)	hypothetical; PP_01390	0
6	complement(143408..143608)	PHAGE_Lactob_Sha1_NC_019489: hypothetical protein; PP_01391; phage(gi418489828)	2.59E-32
7	complement(143766..143996)	hypothetical; PP_01392	0
8	complement(144037..144127)	tRNA	0
9	complement(144162..144281)	hypothetical; PP_01393	0
10	complement(144317..144517)	PHAGE_Lactob_Sha1_NC_019489: hypothetical protein; PP_01394; phage(gi418489829)	1.38E-35
11	complement(144532..145410)	PHAGE_Staphy_P282_NC_048634: endolysin; PP_01395; phage(gi100033)	1.34E-05
12	complement(145473..145886)	PHAGE_Lactob_LBR48_NC_027990: putative receptor protein; PP_01396; phage(gi937456707)	8.41E-49
13	complement(145893..146219)	PHAGE_Lactob_PLE2_NC_031036: hypothetical protein; PP_01397; phage(gi100029)	8.29E-35
14	146476..146691	hypothetical; PP_01398	0
15	146704..147456	PHAGE_Lactob_Sha1_NC_019489: phage-related antirepressor; PP_01399; phage(gi418489833)	3.53E-74
16	complement(147667..148023)	PHAGE_Staphy_phi7401PVL_NC_020199: hypothetical protein; PP_01400; phage(gi448244650)	3.99E-11
17	148081..148341	hypothetical; PP_01401	0
18	complement(148586..148696)	hypothetical; PP_01402	0
19	148707..148805	hypothetical; PP_01403	0
20	complement(148935..149225)	PHAGE_Klebsi_ST437_OXA245phi4.1_NC_049448: u-spanin; PP_01404; phage(gi100032)	1.12E-09
21	149391..149594	PHAGE_Lactob_Sha1_NC_019489: prophage Lp3 protein 4-like protein; PP_01405; phage(gi418489838)	2.69E-07
22	149607..149855	PHAGE_Lactob_Sha1_NC_019489: hypothetical protein; PP_01406; phage(gi418489839)	1.35E-39

23	149858..150058	PHAGE_Lactob_Sha1_NC_019489: hypothetical protein; PP_01407; phage(gi418489840)	2.30E-37
24	150064..150222	PHAGE_Lactob_Sha1_NC_019489: hypothetical protein; PP_01408; phage(gi418489841)	6.74E-13
25	150514..150771	hypothetical; PP_01409	0
26	150873..151670	PHAGE_Lactob_Lj928_NC_005354: hypothetical protein; PP_01410; phage(gi41179305)	1.33E-70
27	151748..152455	PHAGE_Lactob_Sha1_NC_019489: DNA replication protein; PP_01411; phage(gi418489789)	9.33E-173
28	152591..152899	PHAGE_Lactob_Sha1_NC_019489: hypothetical protein; PP_01412; phage(gi418489790)	4.07E-66
29	153132..153602	PHAGE_Lactob_Sha1_NC_019489: phage transcriptional activator RinA; PP_01413; phage(gi418489798)	6.77E-83
30	154296..155084	hypothetical; PP_01414	0
31	155244..155414	PHAGE_Lactob_Sha1_NC_019489: HNH nuclease; PP_01415; phage(gi418489799)	1.38E-32
32	155425..155895	PHAGE_Lactob_Sha1_NC_019489: restriction endonuclease; PP_01416; phage(gi418489800)	9.45E-106
33	155901..156170	hypothetical; PP_01417	0
34	156188..156439	hypothetical; PP_01418	0
35	156626..157084	PHAGE_Lactob_Sha1_NC_019489: P27 family phage terminase small subunit; PP_01419; phage(gi418489801)	4.82E-104
36	157094..158992	PHAGE_Lactob_Sha1_NC_019489: phage terminase-like protein large subunit; PP_01420; phage(gi418489802)	0
37	159048..159176	PHAGE_Lactob_Sha1_NC_019489: phage head-tail joining protein; PP_01421; phage(gi418489803)	1.23E-20
38	159179..160372	PHAGE_Lactob_Sha1_NC_019489: phage portal protein; PP_01422; phage(gi418489804)	0
39	160422..161105	PHAGE_Lactob_Sha1_NC_019489: protease subunit of ATP-dependent Clp protease; PP_01423; phage(gi418489805)	1.11E-153

40	161156..162337	PHAGE_Lactob_Sha1_NC_019489: HK97 family phage major capsid protein; PP_01424; phage(gi418489806)	0
41	162410..162742	PHAGE_Lactob_Sha1_NC_019489: phage protein DNA packaging protein; PP_01425; phage(gi418489807)	1.52E-63
42	162924..163079	PHAGE_Lactob_Lc_Nu_NC_007501: putative head-tail joining protein; PP_01426; phage(gi77999992)	1.87E-06
43	163082..163489	PHAGE_Strept_DT1_NC_002072: putative tail component protein; PP_01427; phage(gi9632427)	8.33E-35
44	163609..163869	PHAGE_Strept_Sfi21_NC_000872: putative tail component protein; PP_01428; phage(gi9632947)	2.96E-10
45	163885..164538	PHAGE_Lactob_phiAT3_NC_005893: putative major tail protein; PP_01429; phage(gi48697269)	5.67E-24
46	164597..164989	hypothetical; PP_01430	0
47	165028..165219	hypothetical; PP_01431	0
48	165251..170281	PHAGE_Strept_DT1_NC_002072: putative tail component protein; PP_01432; phage(gi29165636)	0
49	170422..172128	PHAGE_Lactob_Sha1_NC_019489: minor structural protein gp75-like protein; PP_01433; phage(gi418489815)	0
50	172197..174611	PHAGE_Lactob_Sha1_NC_019489: minor structural protein gp89-like protein; PP_01434; phage(gi418489816)	0
51	174628..177174	PHAGE_Lactob_Sha1_NC_019489: tail fiber; PP_01435; phage(gi418489817)	0
52	177179..177418	PHAGE_Lactob_Sha1_NC_019489: hypothetical protein; PP_01436; phage(gi418489818)	1.28E-39
53	177567..177926	PHAGE_Lactob_Sha1_NC_019489: prophage Lp2 protein 53-like protein; PP_01437; phage(gi418489820)	4.31E-58
54	177938..179110	PHAGE_Lactob_Sha1_NC_019489: endolysin; PP_01438; phage(gi418489821)	0
55	179143..179373	PHAGE_Lactob_Sha1_NC_019489: phage-related holin; PP_01439; phage(gi418489822)	6.73E-47

56	179386..179916	PHAGE_Lactob_Sha1_NC_019489: hypothetical protein; PP_01440; phage(gi418489823)	1.01E-74
57	180732..180745	attR	0
**Region 3, intact, total 57 CDS**			
1	44..55	attL	0
2	complement(66..1196)	PHAGE_Lactob_Sha1_NC_019489: phage integrase; PP_03041; phage(gi418489824)	1.32E-112
3	complement(1345..2265)	PHAGE_Cyanop_P_TIM40_NC_028663: Dam; PP_03042; phage(gi966198395)	5.01E-14
4	complement(2307..4139)	hypothetical; PP_03043	0
5	complement(4263..4886)	hypothetical; PP_03044	0
6	complement(4978..5409)	PHAGE_Lactob_phig1e_NC_004305: hypothetical protein; PP_03045; phage(gi23455774)	3.56E-38
7	complement(5419..5805)	PHAGE_Lactob_phig1e_NC_004305: repressor; PP_03046; phage(gi23455773)	3.90E-26
8	6112..6321	PHAGE_Lactob_phiadh_NC_000896: hypothetical protein; PP_03047; phage(gi9633012)	1.78E-11
9	6325..6531	hypothetical; PP_03048	0
10	6594..6866	PHAGE_Lactob_Sha1_NC_019489: hypothetical protein; PP_03049; phage(gi418489836)	1.14E-38
11	6885..6968	hypothetical; PP_03050	0
12	6983..7432	PHAGE_Lister_2389_NC_003291: Gp40 protein; PP_03051; phage(gi17488545)	4.43E-47
13	7588..7773	hypothetical; PP_03052	0
14	7781..7915	hypothetical; PP_03053	0
15	7917..8021	hypothetical; PP_03054	0
16	8033..8497	PHAGE_Lactob_LfeSau_NC_029068: hypothetical protein; PP_03055; phage(gi985757759)	2.91E-38
17	8614..9873	PHAGE_Lactob_LfeSau_NC_029068: helicase; PP_03056; phage(gi985757760)	0

18	10069..10773	PHAGE_Strept_phiARI0460_1_NC_031913: holin; PP_03057; phage(gi100034)	3.36E-52
19	10776..11375	PHAGE_Lactob_LfeSau_NC_029068: hypothetical protein; PP_03058; phage(gi985757762)	5.23E-23
20	11446..12240	PHAGE_Lactob_LfeSau_NC_029068: DNA primase; PP_03059; phage(gi985757763)	4.99E-87
21	12321..13511	PHAGE_Lactob_LfeSau_NC_029068: hypothetical protein; PP_03060; phage(gi985757764)	1.67E-161
22	13769..14101	PHAGE_Lactob_LfeSau_NC_029068: DNA nuclease; PP_03061; phage(gi985757765)	1.14E-47
23	14121..14288	PHAGE_Lactob_Sha1_NC_019489: hypothetical protein; PP_03062; phage(gi418489791)	9.63E-10
24	14291..14458	hypothetical; PP_03063	0
25	14471..14887	PHAGE_Lactob_SA_C12_NC_047760: portal protein; PP_03064; phage(gi100069)	1.82E-43
26	14960..15415	PHAGE_Lactob_Lb_NC_047983: hypothetical protein; PP_03065; phage(gi100030)	3.49E-73
27	15454..15615	PHAGE_Lactob_Sha1_NC_019489: LP1-like protein; PP_03066; phage(gi418489797)	1.73E-12
28	15732..16040	hypothetical; PP_03067	0
29	16042..16464	hypothetical; PP_03068	0
30	16516..17217	hypothetical; PP_03069	0
31	17495..17911	hypothetical; PP_03070	0
32	18234..18566	PHAGE_Lactob_phig1e_NC_004305: hypothetical protein; PP_03071; phage(gi254854752)	9.41E-11
33	complement(18607..18843)	hypothetical; PP_03072	0
34	18888..19130	hypothetical; PP_03073	0
35	19148..19390	hypothetical; PP_03074	0
36	19499..19786	PHAGE_Staphy_vB_SpsS_QT1_NC_048192: methyltransferase type 11; PP_03075; phage(gi100001)	1.18E-19

37	19861..21462	PHAGE_Bacill_vB_BhaS_171_NC_030904: hypothetical protein; PP_03076; phage(gi100006)	1.34E-180
38	21481..22623	PHAGE_Staphy_P954_NC_013195: HK97 family phage portal protein; PP_03077; phage(gi257136404)	2.19E-115
39	22670..23362	PHAGE_Clostr_phiCTC2B_NC_030951: holin; PP_03078; phage(gi100034)	1.34E-53
40	23383..24561	PHAGE_Lister_LP_101_NC_024387: major capsid protein; PP_03079; phage(gi658607815)	6.16E-123
41	24700..25005	PHAGE_Lister_LP_HM00113468_NC_049900: tail tip protein; PP_03080; phage(gi100054)	8.99E-13
42	25163..25375	PHAGE_Lister_LP_HM00113468_NC_049900: tail assembly protein; PP_03081; phage(gi100053)	9.91E-09
43	25381..25779	PHAGE_Lister_LP_HM00113468_NC_049900: tail fiber protein; PP_03082; phage(gi100052)	1.93E-16
44	25794..26198	PHAGE_Lister_LP_101_NC_024387: hypothetical protein; PP_03083; phage(gi658607820)	1.27E-18
45	26213..26824	PHAGE_Staphy_6ec_NC_024355: major tail protein; PP_03084; phage(gi658310345)	1.09E-23
46	26903..27232	hypothetical; PP_03085	0
47	27340..27477	hypothetical; PP_03086	0
48	27496..32040	PHAGE_Lactob_PL_1_NC_022757: tape measure; PP_03087; phage(gi557308006)	0
49	32044..32865	PHAGE_Entero_phiFL1A_NC_013646: tail protein; PP_03088; phage(gi281416378)	2.41E-35
50	32885..36811	PHAGE_Strept_P9_NC_009819: hypothetical protein; PP_03089; phage(gi157311177)	7.23E-90
51	36834..37319	PHAGE_Brocho_BL3_NC_015254: gp20; PP_03090; phage(gi327409412)	5.37E-09
52	37321..37755	hypothetical; PP_03091	0

53	37772..38155	PHAGE_Lactob_LfeSau_NC_029068: hypothetical protein; PP_03092; phage(gi985757745)	7.21E-37
54	38158..38352	PHAGE_Lactob_phijl1_NC_006936: hypothetical protein; PP_03093; phage(gi62327114)	7.55E-08
55	38355..38636	PHAGE_Lactob_phig1e_NC_004305: holin; PP_03094; phage(gi23455817)	6.93E-06
56	38678..39529	PHAGE_Entero_EF62phi_NC_017732: endolysin type Endo-N-acetylmuramidase; PP_03095; phage(gi384519788)	2.39E-39
57	39629..39640	attR	0
**Region 4, questionable, total 26 CDS**			
1	105..119	attL	0
2	complement(522..722)	PHAGE_Vibrio_1.026.O._10N.222.49.C7_NC_049430: portal protein; PP_03387; phage(gi100069)	1.27E-09
3	complement(855..1214)	PHAGE_Lactob_PL_1_NC_022757: transcriptional regulator; PP_03388; phage(gi557308042)	2.28E-06
4	complement(1359..1625)	PHAGE_Entero_IME_EFm5_NC_028826: head-tail joining protein; PP_03389; phage(gi971746942)	1.98E-09
5	1694..1765	tRNA	0
6	complement(1824..1934)	hypothetical; PP_03390	0
7	complement(2035..3591)	PHAGE_Entero_EFRM31_NC_015270: major capsid protein; PP_03391; phage(gi327198115)	2.64E-43
8	complement(3605..4711)	PHAGE_Staphy_StB20_NC_019915: portal HK97 family; PP_03392; phage(gi431809767)	1.73E-60
9	complement(4712..4843)	hypothetical; PP_03393	0
10	complement(4866..6401)	PHAGE_Strept_315.2_NC_004585: hypothetical protein; PP_03394; phage(gi28876230)	1.13E-120
11	complement(6479..6559)	hypothetical; PP_03395	0
12	complement(6566..7039)	PHAGE_Entero_EFRM31_NC_015270: terminase small subunit; PP_03396; phage(gi327198110)	3.53E-15
13	7182..7376	hypothetical; PP_03397	0

14	7381..7542	hypothetical; PP_03398	0
15	complement(7732..8109)	PHAGE_Staphy_Pvl108_NC_008689: phage endonuclease; PP_03399; phage(gi119443688)	9.56E-23
16	complement(8114..8455)	PHAGE_Lactob_Sha1_NC_019489: phage head-tail adaptor; PP_03400; phage(gi418489808)	5.15E-16
17	complement(8465..8647)	hypothetical; PP_03401	0
18	complement(8671..9090)	hypothetical; PP_03402	0
19	complement(9235..10617)	PHAGE_Staphy_vB_SpsS_QT1_NC_048192: minor tail protein; PP_03403; phage(gi100055)	1.56E-81
20	complement(10629..11330)	PHAGE_Lactoc_bIL310_NC_002669: hypothetical protein; PP_03404; phage(gi13095885)	2.48E-18
21	complement(11426..11680)	hypothetical; PP_03405	0
22	complement(11801..11908)	hypothetical; PP_03406	0
23	complement(11951..12130)	PHAGE_Lactob_Sha1_NC_019489: prophage Lp3 protein 4-like protein; PP_03407; phage(gi418489838)	3.58E-30
24	12269..12922	PHAGE_Faecal_FP_Taranis_NC_047914: hypothetical protein; PP_03408; phage(gi100084)	9.98E-09
25	13001..14155	PROPHAGE_Oceano_HTE831: integrase; PP_03409; phage(gi23097608)	2.99E-85
26	14324..14338	attR	0
